# Formation of Chromophores from *cis*-Pinonaldehyde Aged in Highly Acidic Conditions

**DOI:** 10.1021/jacs.3c14177

**Published:** 2024-04-19

**Authors:** Cynthia Wong, Jessica E. Pazienza, Scott D. Rychnovsky, Sergey A. Nizkorodov

**Affiliations:** Department of Chemistry, University of California, Irvine, Irvine, California 92697-2025, United States

## Abstract

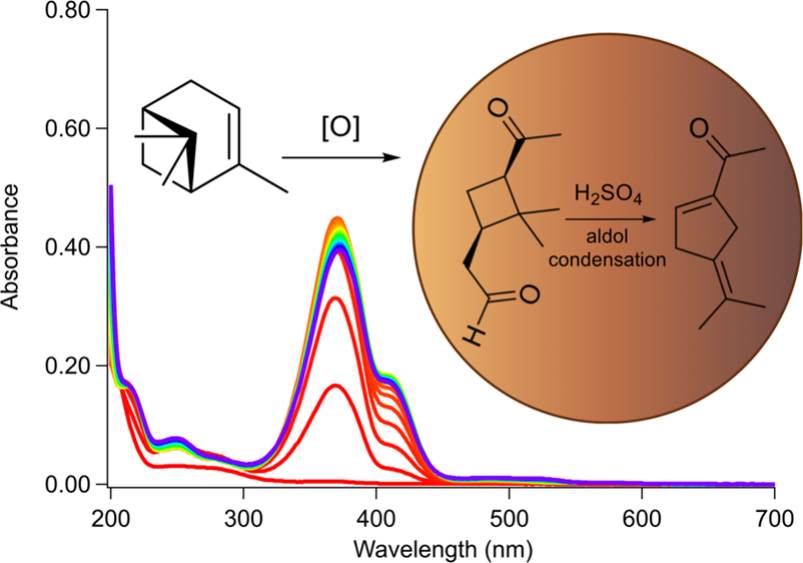

Sulfuric acid in the atmosphere can participate in acid-catalyzed and acid-driven reactions, including those within secondary organic aerosols (SOA). Previous studies have observed enhanced absorption at visible wavelengths and significant changes in the chemical composition when SOA was exposed to sulfuric acid. However, the specific chromophores responsible for these changes could not be identified. The goals of this study are to identify the chromophores and determine the mechanism of browning in highly acidified α-pinene SOA by following the behavior of specific common α-pinene oxidation products, namely, *cis*-pinonic acid and *cis*-pinonaldehyde, when they are exposed to highly acidic conditions. The products of these reactions were analyzed with ultra-performance liquid chromatography coupled with photodiode array spectrophotometry and high-resolution mass spectrometry, UV–vis spectrophotometry, and nuclear magnetic resonance spectroscopy. *cis*-Pinonic acid (**2**) was found to form homoterpenyl methyl ketone (**4**), which does not absorb visible radiation, while *cis*-pinonaldehyde (**3**) formed weakly absorbing 1-(4-(propan-2-ylidene)cyclopent-1-en-1-yl)ethan-1-one (**5**) and 1-(4-isopropylcyclopenta-1,3-dien-1-yl)ethan-1-one (**6**) via an acid-catalyzed aldol condensation. This chemistry could be relevant for environments characterized by high sulfuric acid concentrations, for example, during the transport of organic compounds from the lower to the upper atmosphere by fast updrafts.

## Introduction

Organic compounds make up a substantial portion of mass within atmospheric particulate matter, ranging from 20 to 90%.^1−3^ A subset of these organics known as “brown carbon” comprises compounds capable of absorbing near-UV and visible radiation.^4^ These light-absorbing compounds directly and indirectly impact the radiative energy budget in the Earth’s atmosphere, resulting in diminished visibility, worsening air quality, and negative impacts on regional climate.^4−7^ However, the mechanisms of formation and the structures of these compounds remain poorly defined.

Brown carbon aerosols can be of primary origin, such as those directly emitted from fossil fuels and biomass burning.^4^ Secondary organic aerosol (SOA) produced by the oxidation of aromatic compounds also contributes to brown carbon.^4^ SOA produced by the oxidation of biogenic organics generally does not absorb visible radiation.^8,9^ However, biogenic SOA can produce brown carbon by multiphase aging processes occurring during atmospheric transport.^4,10−16^ These processes, which can take days under ambient atmospheric conditions, alter both the chemical composition and optical properties of organic aerosol particles.^17−20^ Of particular interest to this work is the formation of brown carbon promoted by particle acidity, which typically ranges between pH −1 to 5 in atmospheric particles.^21^ Previous studies have observed brown carbon resulting from reactions of organic compounds in acidic solutions and acid-catalyzed reactions in aqueous solutions.^14,22−30^ However, the structures of these compounds and their formation mechanisms remain unknown.

**Figure 1:**
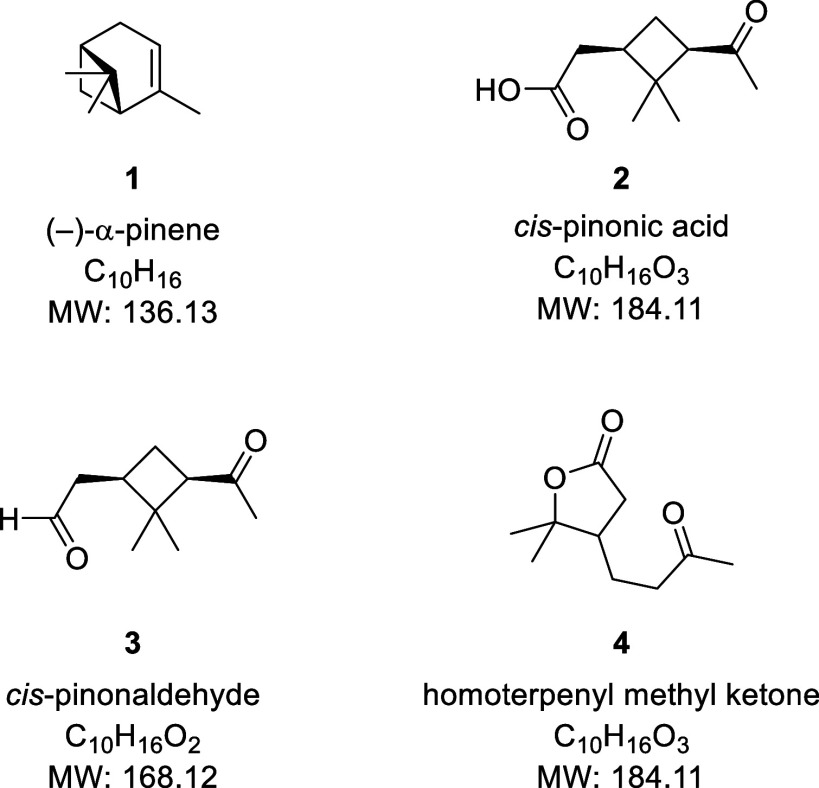
Structures of (−)-α-pinene (**1**), *cis*-pinonic acid (**2**), *cis*-pinonaldehyde (**3**), and homoterpenyl methyl ketone (**4**).

One of the largest contributors to SOA is α-pinene (**1**, Figure 1), with estimated emissions of ∼66 Tg/year.^31^ Both (+) and (−) enantiomers of α-pinene are emitted by vegetation.^32^ Once emitted, α-pinene can react with O_3_, •OH, or •NO_3,_ which leads to the production of multifunctional organic compounds containing carbonyl, carboxyl, hydroxyl, peroxide, and other functional groups. *cis*-Pinonic acid (**2**, Figure 1) and *cis*-pinonaldehyde (**3**, Figure 1) are two major products occurring from ozonolysis and •OH-oxidation of α-pinene (**1**).^33,34^*cis*-Pinonic acid (**2**) is a C_10_ ketocarboxylic acid with a rigid, four-membered carbon ring decorated by an acetyl and a carboxyl group on opposing ends of the cyclobutane core, as shown in Figure 1. *cis*-Pinonaldehyde (**3**) has a similar structure but with a formyl group in place of the carboxyl group, making it a ketoaldehyde. These molecules are both semivolatile compounds that can exist in either the gas or particle phase (with lower temperature and higher particulate matter concentrations favoring the particle phase).^35^ The properties and atmospheric fates of *cis-*pinonic acid and *cis-*pinonaldehyde have been studied extensively.^36−42^

Previous work explored chemical changes that may occur upon exposure of α-pinene SOA to highly acidic conditions. One study found that the evaporation of bulk biogenic and anthropogenic SOA solutions, including that of α-pinene derived SOA, in the presence of sulfuric acid, resulted in enhanced mass-normalized absorption coefficient at visible wavelengths and significant changes in the chemical composition, including organosulfate formation.^11,43^ Work by Wong et al. offered a more careful control of pH and reported facile formation of light-absorbing compounds and organosulfates at highly acidic conditions (pH −0.86 and pH −1.08) from α-pinene ozonlysis.^30^ However, these studies could not identify the specific chromophores responsible for the enhanced light absorption. The aim of this work is to determine the mechanism of browning in highly acidified α-pinene SOA by following the behavior of specific common α-pinene oxidation products, namely, *cis*-pinonic acid and *cis*-pinonaldehyde, when they are exposed to highly acidic conditions. Our results suggest that *cis*-pinonic acid undergoes an acid-catalyzed isomerization when aged in highly acidic conditions, and the products do not absorb visible radiation. In contrast, *cis-*pinonaldehyde reacts with the acid to form two chromophores via intramolecular aldol condensation. These chromophores may be responsible for the browning of acid-aged SOA observed in previous studies.

## Experimental Methods and Materials

### Aging in Sulfuric Acid

For the initial tests, *cis*-pinonic acid and *cis*-pinonaldehyde were each dissolved in 10 M H_2_SO_4_, resulting in an effective pH of −1.08, with dissolution in water serving as a control. The pH values cited in this work correspond to the negative logarithm of the molality of H^+^, which is estimated using the extended aerosol inorganic model I (E-AIM).^44−46^ Once it was confirmed that there were significant changes in the composition and optical properties of the acidified sample, additional experiments were performed at varying acidities. In all cases, an aliquot of the stock *cis*-pinonic acid or *cis*-pinonaldehyde with a concentration of 2000 μg/mL was added to a 4 mL aqueous solution containing H_2_SO_4_ (Table S1), resulting in a mass concentration of 35–70 μg/mL. To conduct NMR analysis, it was necessary to scale up the reaction to generate sufficient material for purification and subsequent analysis, as explained in detail in the SI. All samples were neutralized with sodium carbonate before disposal.

### Mass Spectrometry Analysis

Thermo Scientific Vanquish Horizon ultra-performance liquid chromatography (UPLC) instrument coupled to a Vanquish Horizon photodiode array (PDA) spectrophotometer and a Thermo Scientific Q Exactive Plus Orbitrap high-resolution mass spectrometer were used to examine the chemical composition of the solution before and after aging. UPLC separation was carried out on a Waters HSS T3 column, 150 × 2.1 mm, with 1.8 μm particles, with the column temperature set to 30 °C and a flow rate of 0.3 mL/min. The mobile phase consisted of water (eluent A) and acetonitrile (eluent B), each containing 0.1% formic acid. The gradient elution was programmed as follows: 0–3 min 95% eluent A; 3–14 min linear ramp to 95% eluent B; 14–16 min hold at 95% eluent B, 16–22 min return to 95% eluent A. The sample injection volume was 10 μL. It should be noted that repeated injections of these highly acidic samples led to a degradation of the UPLC column, but we intentionally did not neutralize the samples before the injection to avoid altering the chemistry. The mass spectrometer had an electrospray ionization (ESI) operated in either positive (+) or negative (−) ion mode with a spray voltage of 2.5 kV and a resolving power of *m*/Δ*m* = 1.4 × 10^5^.

### Spectroscopic Measurements

A UV–vis spectrophotometer (Shimadzu UV-2450) was used to record absorption spectra over the 200–700 nm range with 1 nm resolution to monitor the formation of light-absorbing compounds over time. Aliquots of the samples were added to a 1 cm quartz cuvette and then capped to prevent evaporation. The spectrophotometer was programmed to collect a spectrum every 15 min for 24 h.

## Results and Discussion

### Ultra-Performance Liquid Chromatography

**Figure 2:**
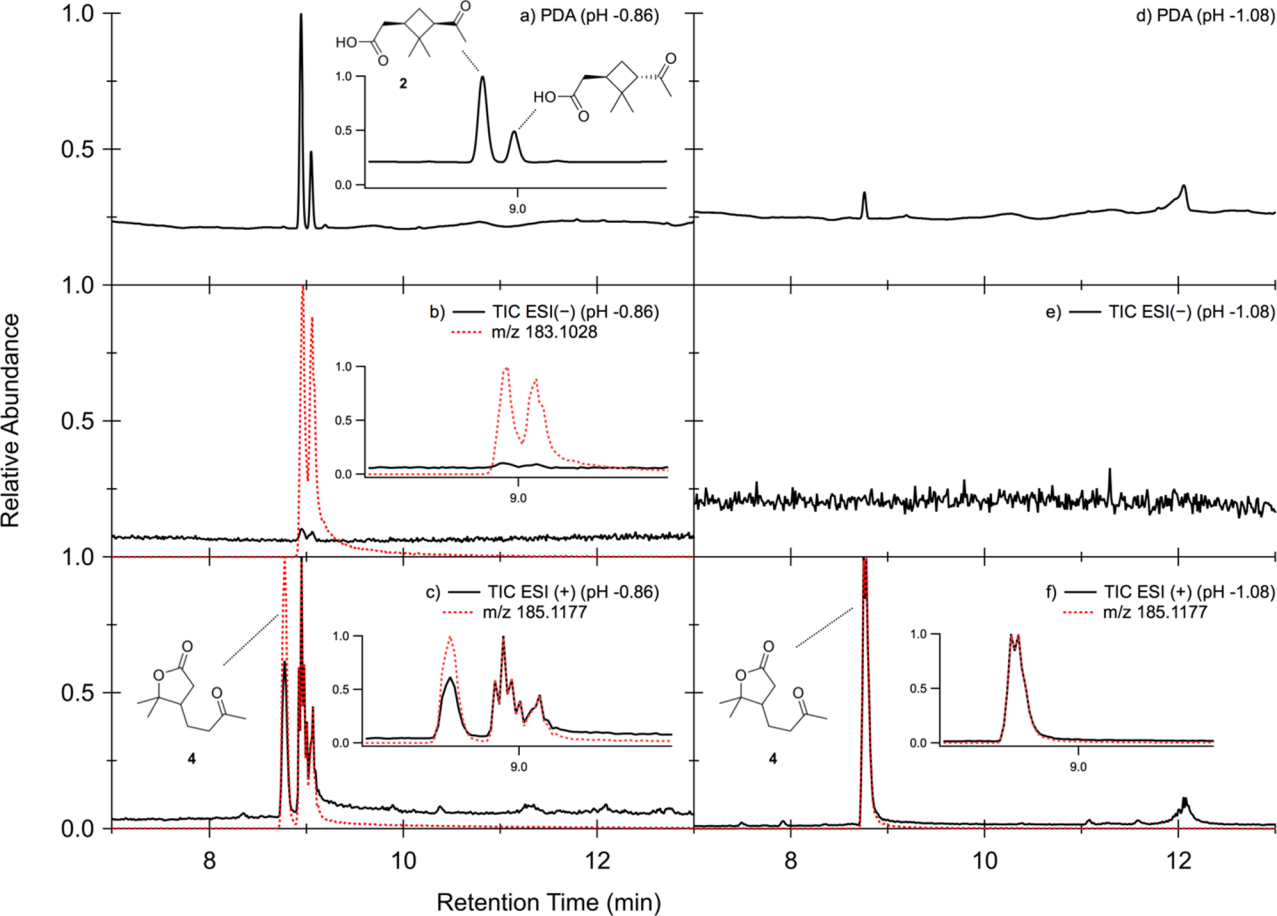
UPLC chromatograms of *cis*-pinonic acid aged in 5.6 M H_2_SO_4_ (pH −0.86) and 10 M H_2_SO_4_ (pH −1.08) for 2 days observed in the ESI(−) mode (b,e), ESI(+) mode (c,f), and with the PDA (a,d). Panels b–f also show SIC for *m*/*z* 183.1028 and *m*/*z* 185.1177, respectively. The PDA chromatograms were shifted 0.06 min to account for the time delay between the PDA and MS detectors. All chromatograms were normalized based on the maximum peak intensity of their respective data set.

The total ion chromatograms (TIC) and PDA (190–690 nm) chromatograms of *cis*-pinonic acid aged for 2 days in H_2_O (control, pH 4.3) and 0.52 mM H_2_SO_4_ (pH 3.00) are shown in Figure S1, while the data for the samples aged in 5.6 M H_2_SO_4_ (pH −0.86), and 10 M H_2_SO_4_ (pH −1.08) are shown in Figure 2. A single major peak at 8.96 min was detected in the PDA (Figure S1a,d), negative ion mode TIC (Figure S1b,e), and positive ion mode TIC (Figure S1c,f) chromatograms of *cis*-pinonic acid aged in H_2_O (pH 4.3) and 0.52 mM H_2_SO_4_ (pH 3.00). The PDA signal corresponds to weak absorption of *cis*-pinonic acid due to the *n* → π* and π → π* transitions in the carbonyl group. The TIC signal is dominated by protonated (positive ion mode) or deprotonated (negative ion mode) *cis*-pinonic acid. The nearly identical chromatograms of *cis*-pinonic acid aged in H_2_O (pH 4.3) and 0.52 mM H_2_SO_4_ (Figure S1) indicate that it does not undergo acid-catalyzed reactions under moderately acidic conditions (pH 3.0).

However, when *cis*-pinonic acid was aged in highly acidic conditions, the chromatograms revealed chemical changes driven by acid catalysis, in agreement with previous observations by Arcus and Bennett.^47^ In Figure 2a, which shows the PDA chromatograms of *cis*-pinonic acid aged in 5.6 M H_2_SO_4_ (pH −0.86), there are two peaks at 8.95 and 9.06 min. The negative ion mode TIC and selected ion chromatogram (SIC) for *m*/*z* 183.1028 in Figure 2b also have two peaks at these elution times. The peak at 9.06 min is likely *trans*-pinonic acid, as cited in Arcus and Bennett,^47^ who observed that *cis*-pinonic acid can isomerize to *trans*-pinonic acid under highly acidic conditions. The two isomers also appear in positive ion modes TIC and SIC for *m*/*z* 185.1177 (Figure 2c). However, the positive ion mode chromatograms also have an additional peak that elutes at 8.78 min. This can be assigned to the homoterpenyl methyl ketone (**4**, Figure 1).^47−49^ When *cis*-pinonic acid is aged in 10 M H_2_SO_4_ (pH −1.08), Figure 2f shows that all of the initial starting material has been converted to a homoterpenyl methyl ketone.

Experiments were then repeated for *cis*-pinonaldehyde. Positive ion mode ESI proved to be more useful at detecting changes in chemical composition as opposed to negative ion mode, which is better suited for carboxylic acids. From here on out, the data described in the text refer to the positive ion mode.

Samples in which *cis*-pinonaldehyde was aged in H_2_O (control, pH 4.3), 0.52 mM H_2_SO_4_ (pH 3.00), and 1.0 M H_2_SO_4_ (pH −0.01) each had two dominant peaks, as shown in Figure S2. The smaller peak at 8.94 min corresponds to *cis*-pinonic acid, an impurity that stems from the synthesis of *cis*-pinonaldehyde, while the peak at 9.77 min corresponds to *cis*-pinonaldehyde. The same peaks occur in the 0.52 mM H_2_SO_4_ (pH 3.00), and 1.0 M H_2_SO_4_ (pH −0.01) samples, and there are no additional peaks, which indicates that aging the starting material at these conditions does not promote acid-catalyzed reactions.

**Figure 3:**
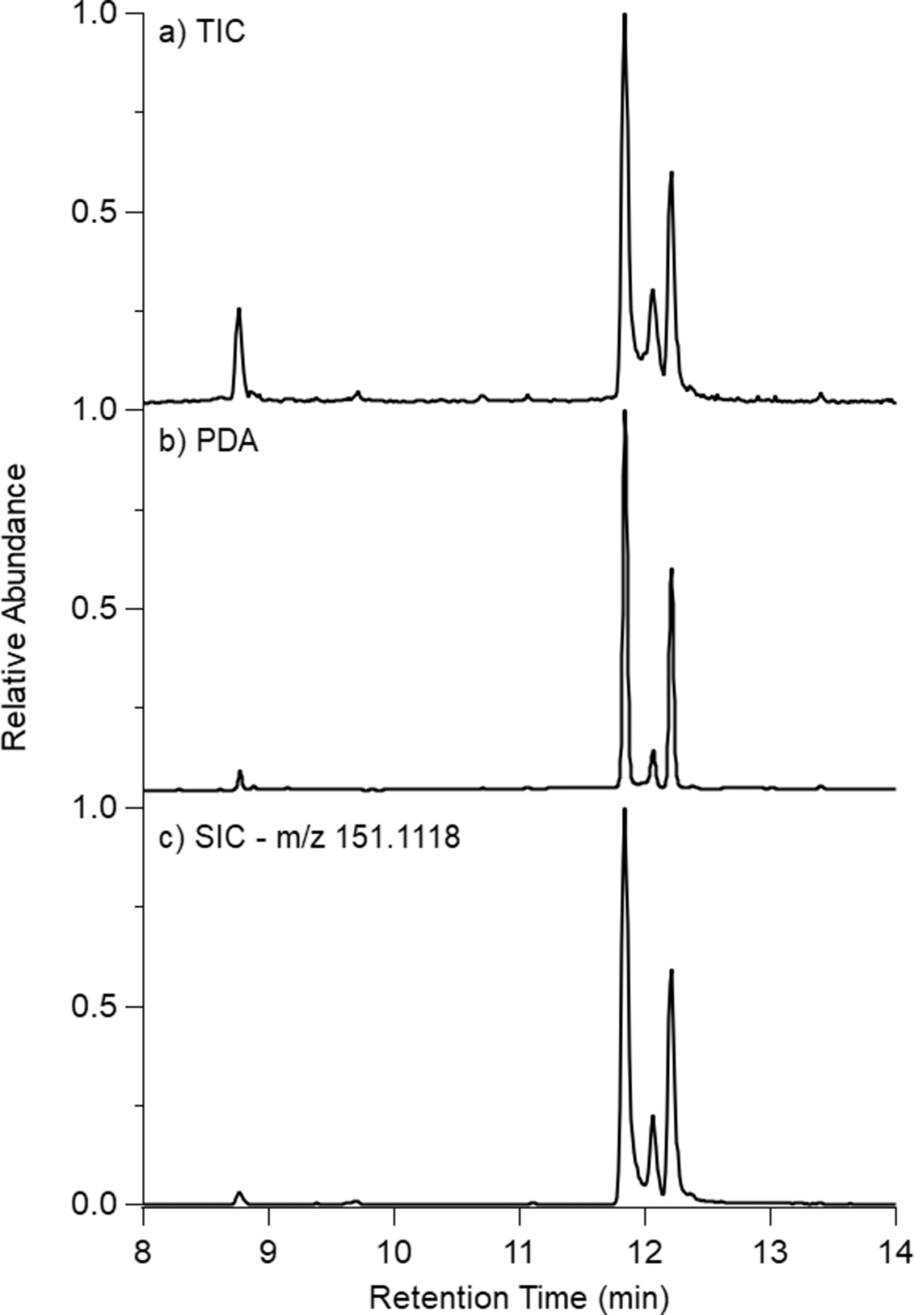
UPLC chromatograms of *cis*-pinonaldehyde aged in 10 M H_2_SO_4_ observed in the ESI(+) mode for TIC (a), PDA (b), and SIC (*m*/*z* 151.1117) (c). The PDA chromatogram was shifted by 0.06 min to account for the time delay between the PDA and MS detectors.

However, the TIC (Figure 3a) of the sample in which *cis*-pinonaldehyde was aged in highly acidic conditions (10 M H_2_SO_4_) implies alternate chemical pathways. There are four significant peaks under these aging conditions at retention times (RTs) of 8.77, 11.85, 12.07, and 12.22 min. Additionally, in this chromatogram, the peak corresponding to *cis*-pinonaldehyde is absent, indicating that all *cis*-pinonaldehyde was consumed. The peak at 8.77 min corresponds to the acid-catalyzed isomerization product of the *cis*-pinonic acid impurity, homoterpenyl methyl ketone (**4**). The remaining three peaks correspond to the same dominant ion in the mass spectrum (*m*/*z* 151.1117, C_10_H_15_O^+^), suggesting that these compounds are isomers (Figure 3c). The same peaks appear in the PDA chromatogram of the sample, shown in Figure 3b, which shows that these compounds are the chromophores of interest.

### Kinetic Analysis

The absorption spectra of solutions of *cis*-pinonic acid and *cis*-pinonaldehyde were recorded with the UV–vis spectrophotometer every 15 min over 24 h under various aging conditions. As no measurable absorbance evolved in the near-UV and visible ranges, the absorption spectra of *cis*-pinonic acid are only discussed in the SI section (Figure S3 and adjacent text).

**Figure 4:**
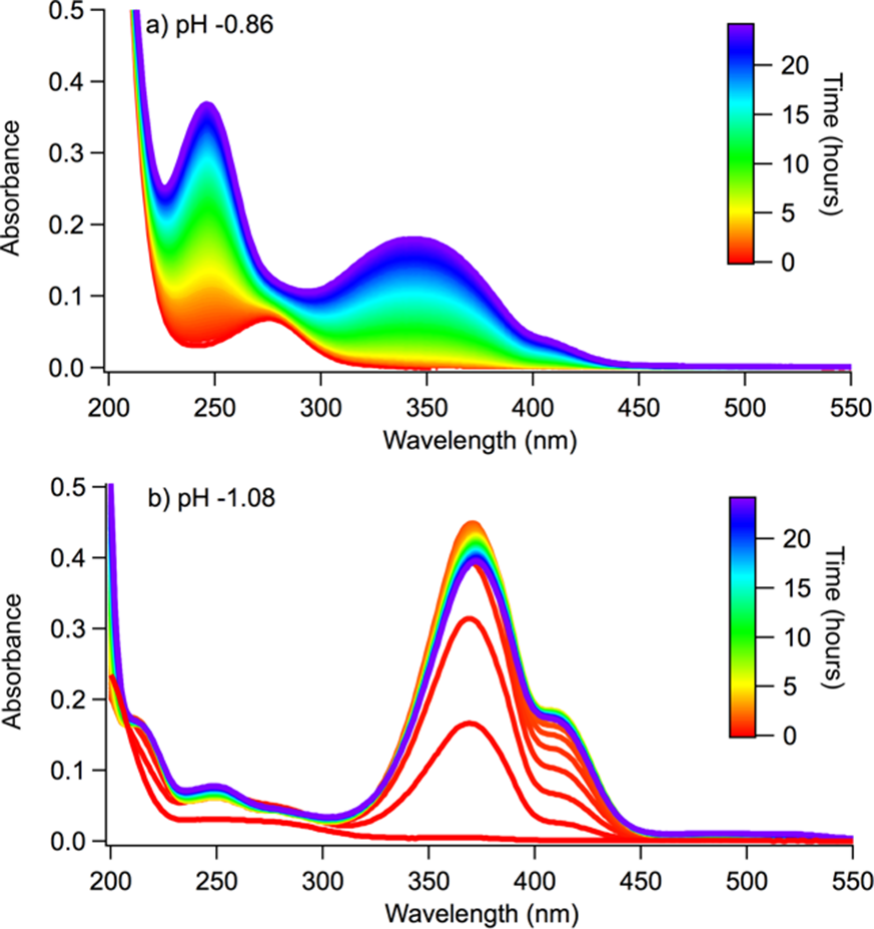
Absorption spectra of *cis*-pinonaldehyde aged in (a) 5.6 M (pH −0.86) and (b) 10 M (pH −1.08) H_2_SO_4_. Each spectrum was collected every 15 min over 24 h.

The absorption spectra of *cis*-pinonaldehyde aged in 5.6 M (pH −0.86) and 10 M (pH −1.08) H_2_SO_4_ are shown in Figure 4. In Figure 4a, two dominant peaks increase as a function of time at 246 and 355 nm. Additionally, the absorption spectrum of *cis*-pinonaldehyde aged in 10 M (pH −1.08) H_2_SO_4_ shows five main peaks (Figure 4b) at 215, 251, 370, 415, and 500 nm. The effective lifetime of browning for each peak within these samples was calculated by assuming pseudo-first-order reactions in the time series fits for each peak, which are outlined in Table 1. At pH −1.08, the effective browning lifetime is on the order of 1 h.

**Table 1:** Effective Lifetimes of Browning for *cis*-Pinonaldehyde Aged in 5.6 M (pH −0.86) and 10 M (pH −1.08) H_2_SO_4_, Calculated by Assuming Pseudo-First-Order Reactions in the Time Series Fits for Each Peak

sample	peak of interest (nm)	lifetime of browning (h)
pH −0.86	246	18 ± 3
	355	69 ± 1
pH −1.08	215	0.47 ± 0.03
	251	0.04 ± 0.03
	370	0.31 ± 0.03
	415	0.69 ± 0.03
	500	2.4 ± 0.2

**Figure 5:**
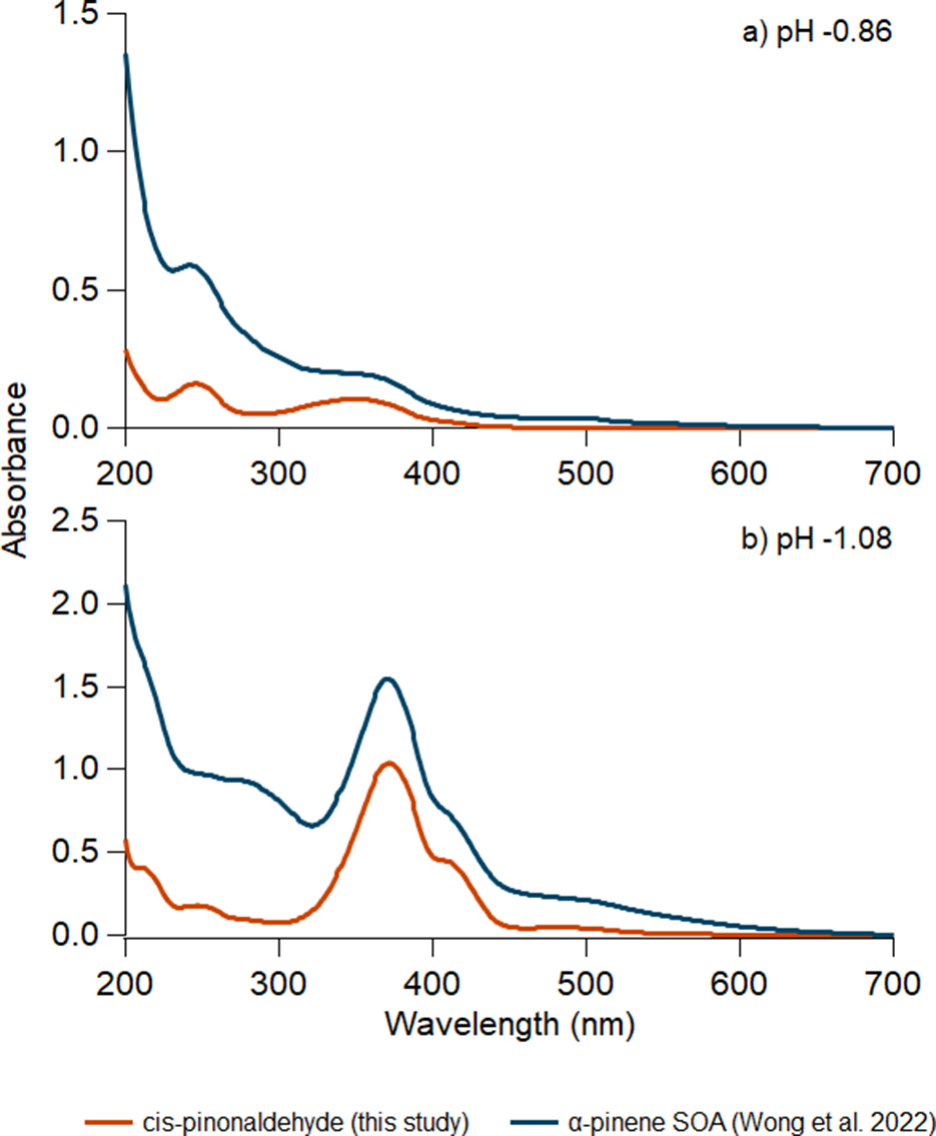
Comparison of the absorption spectra of *cis-*pinonaldehyde (**3**) and SOA prepared by ozonolysis of α-pinene (**1**) after being aged for 2 days in (a) 5.6 M (pH −0.86) and (b) 10 M (pH −1.08) H_2_SO_4_. The SOA data are from Wong et al.^30^

The general shape of the absorption spectrum of aged *cis*-pinonaldehyde is similar to that observed during the aging of α-pinene SOA under the same highly acidic conditions, as shown in Figure 5.^30^ This implies that *cis*-pinonaldehyde should play a major role in the mechanism of browning of acidified α-pinene SOA. The subtle differences between the aged *cis*-pinonaldehyde and α-pinene SOA spectra likely arise from other compounds in the α-pinene SOA that may also produce light-absorbing products upon aging. It is also noted that the absorption of the products of *cis*-pinonaldehyde aged in 10 M H_2_SO_4_ (pH −1.08) reaches a maximum and then starts to decay the longer it ages, indicating that other reactions could take place that consume the chromophore of interest.

## Product Identification

### Purification and Separation of Chromophores

In brief, a crude solution of *cis-*pinonaldehyde aged in 10 M H_2_SO_4_ (pH −1.08) was subjected to aqueous extraction and purification via flash chromatography. Three UV active peaks were observed during column chromatography and are believed to be the three chromophores of interest. The most and least polar of the three were isolated as reasonably pure samples. The second compound could not be isolated owing to its negligible mass fraction of the mixture. The two isolates were analyzed via 1D and 2D nuclear magnetic resonance (NMR) spectroscopy, infrared (IR) spectroscopy, and high-resolution mass spectroscopy (HRMS). Additional hydrogenation experiments were also conducted to better determine the atom connectivity in these compounds.

Note that during the purification and separation process, NaHCO_3_ was added to neutralize the acid and allow for the isolation of the chromophores. It is worth noting that trace amounts of *cis*-pinonic acid were detected in the sample of *cis*-pinonaldehyde, which explains the observed formation of the homoterpenyl methyl ketone. Furthermore, it is plausible that a reaction takes place during the aging process of *cis*-pinonaldehyde, resulting in the generation of *cis*-pinonic acid, which can contribute to the increased abundance of homoterpenyl methyl ketone. We also emphasize that the purification and analysis of the compounds were challenging because of their relatively low yield and poor stability. The chromophores of interest make up only 3% and 8% of the overall mixture and are prone to decomposition at high concentrations and in the presence of oxygen. The following is a summary of the proposed structural information determined by the analysis described below.

### NMR Analysis and Plausible Mechanism

**Figure 6:**
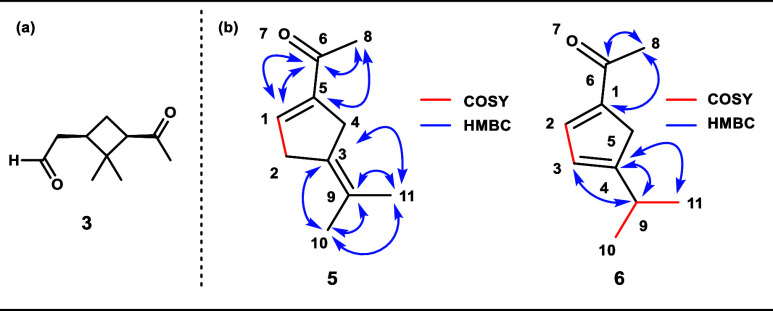
(a) Structure of *cis*-pinonaldehyde (**3**) and (b) structures and diagnostic 2D NMR cross peaks of chromophores **5** and **6**.

NMR analysis was performed to determine the structural information for chromophores **5** and **6**. Diagnostic 2D cross peaks that informed our assignment are shown in Figure 6b.

Compound **5** (Figure 6b) was isolated as a light-yellow oil. Its HRMS spectrum gave the molecular formula C_10_H_14_O, indicating four degrees of unsaturation. Its ^1^H and ^13^C-DEPTQ NMR spectra revealed the existence of two double bonds: one tetrasubstituted (δ_C_ 133.1 (C^3^), 140.5 (C^9^)) and one monosubstituted (δ_H_ 7.16 (H^1^); δ_C_ 140.9 (C^1^), 146.3 (C^5^)). One of these alkenes is part of an enone. Further resonances of three methyl singlets were observed (δ_H_ 2.33 (H^8^), 1.87 (H^11^), and 1.74 (H^10^)), one of which corresponds to a methyl ketone (δ_C_ 197.1 (C^6^), H^8^). Two methylenes account for the total 14 protons (δ_H_ = 2.63 (H^2^), 2.53 (H^4^)). In addition to the carbon signals assigned to the alkenes, the ^13^C-DEPTQ NMR spectra revealed an additional five carbons: three CH_3_ groups (δ_C_ 26.7 (C^8^), 22.0 (H^10^), 21.5(H^11^)) and two CH_2_ groups (δ_C_ 29.3 (C^2^), 27.8 (C^4^)). The aforementioned functionalities—an alkene and an enone—account for three out of the four degrees of unsaturation. Therefore, the fourth hinted at the presence of a ring in this compound. The spatial relationships between all of these fragments were determined with 2D NMR spectroscopy. Cross-peaks in the ^1^H–^1^H correlated spectroscopy (COSY) and heteronuclear multiple bond correlation (HMBC) spectra showed a structural segment of} −CH_2_^2^–CH^1^=C^5^–C^6^(O)–CH_3_^8^. These fragments exhausted the information in the 2D NMR spectra, so we turned to mechanistic insight to inform our analysis of how they are interconnected.

**Scheme 1:**
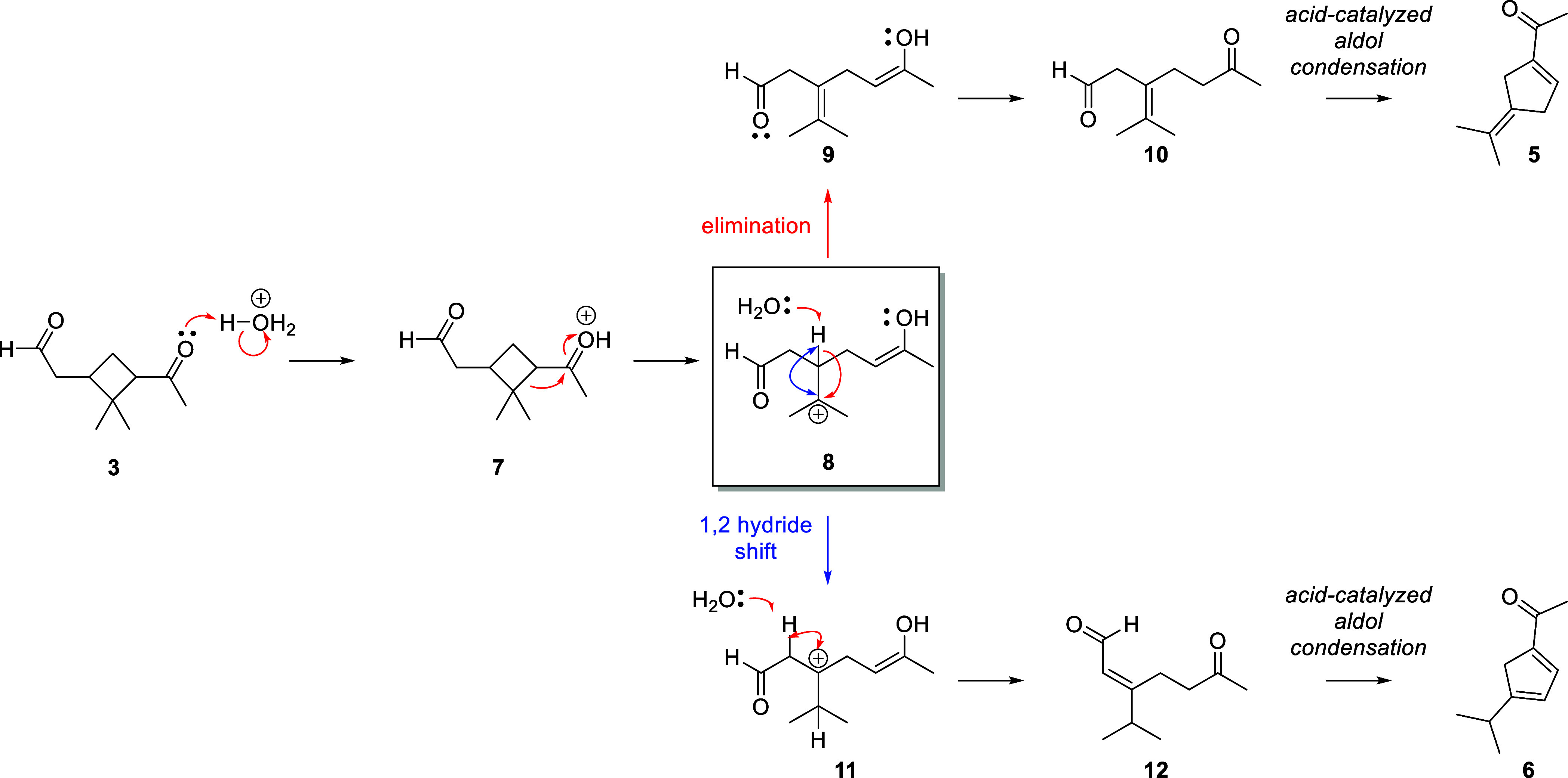
Mechanism of Formation of Compounds **5** and **6** For detailed mechanisms of the acid-catalyzed aldol condensation, refer to Schemes S2 and S3 in the Supporting Information.

Scheme 1 shows a possible mechanism for the formation of compound **5**. Under highly acidic aging conditions, ketone **3** likely exists in its protonated form **7**. This catalyzes a ring opening of the strained cyclobutane core to form the tertiary carbocation **8**. Elimination of the neighboring methine proton would furnish olefin **9**. Cyclopentene **5** could then be formed by an acid-catalyzed intramolecular aldol condensation. Turning to the literature, we found that compound **5** is a known terpene oxidation product, though without consistent and complete spectroscopic characterization reported.^50,51^ Supported by spectroscopic, mechanistic, and literature evidence, we assigned the structure of compound **5** (Figure 6b).

Compound **6** (Figure 6b), obtained as an orange oil, was deduced to have an identical molecular formula to that of compound **5** according to HRMS. The 1D NMR data also closely resembled that of compound **5**, indicating that these two compounds are constitutional isomers. With the scaffold of compound **5** in mind, the following deductions were made. A methyl ketone was observed in both the ^1^H and ^13^C NMR (δ_C_ = 193.7 (C^6^), 26.0 (C^8^); δ_H_ = 2.33–2.30 (H^8^). The NMR spectra also showed an isopropyl group (δ_C_ 30.3 (C^9^) 22.8 (C^10,11^); δ_H_ 2.72 (H^9^), 1.14 (H^10,11^), which we presume was a substituent on the five-membered ring.

The ^13^C spectra of **6** indicated the presence of two alkenes (δ_C_ 145.0 (C^1^), 143.9 (C^2^), 125.0 (C^3^), and 166.1 (C^4^)), with one of them belonging to an enone. The ^1^H NMR revealed two alkene protons (δ_H_ 7.24 (H^2^), 6.26–6.16 (H^3^). These two olefinic proton resonances could correspond to either: one 1,2-disubstituted alkene and one tetrasubstituted alkene, or two trisubstituted alkenes.

From the HMBC and COSY spectra, an enone and a vinylic isopropyl group were pieced together. Unfortunately, at this point, NMR spectroscopy became ambiguous as to the substitution pattern, either 1,2- or 1,3-, around the five-membered ring. However, we reasoned that hydrogenation of this adduct would allow us to differentiate between the two possibilities, given that one hydrogenation adduct is reported in the literature.^52^ Hydrogenation of **6** resulted in a 1:1 mixture of diastereomers that were different from those reported by Hoveyda and co-workers. Therefore, we concluded that the third chromophore with a molecular formula of C_10_H_14_O was the 1,3-disubstituted cyclopentadiene **6** (Figure 6b), which is formed through a mechanism nearly identical to that of chromophore **5** (Scheme 1).

A plausible mechanism is shown in Scheme 1. Ring-opening of **3** gives common cation intermediate **8**. Here, the mechanism diverges from that of **5** with a 1,2-hydride shift to afford **11**. Elimination affords ketoaldehyde **12**, which undergoes a similar aldol condensation as seen in the mechanism for compound **5** to afford compound **6**. Alternatively, acid-catalyzed equilibration between intermediates **10** and **12** and compounds **5** and **6** is possible, but we have not demonstrated this additional mechanism.

## Conclusions and Implications

The composition and optical properties of organic aerosols can undergo significant changes due to acid-catalyzed and acid-driven reactions. The impacts of highly acidic conditions on *cis*-pinonic acid and *cis*-pinonaldehyde, known products of α-pinene oxidation in the atmosphere, were explored by aging these compounds in bulk sulfuric acid solutions with atmospherically relevant acidities. It was found that *cis*-pinonic acid formed homoterpenyl methyl ketone (**4**) while *cis*-pinonaldehyde formed 1-(4-(propan-2-ylidene)cyclopent-1-en-1-yl)ethan-1-one (**5**) and 1-(4-isopropylcyclopenta-1,3-dien-1-yl)ethan-1-one (**6**) under highly acidic conditions. Compounds **5** and **6** are stronger light absorbers than *cis*-pinonaldehyde due to their extended bond conjugation. This will affect the attenuation of incoming sunlight by atmospheric organics, especially in the ultraviolet range. Additionally, they are expected to have higher volatility than pinonaldehyde, so the gas-particle partitioning of organics could be affected, as well.

These findings could be relevant in the context of the upper troposphere and lower stratosphere (UTLS). Aerosols in UTLS are composed of sulfuric acid (40–80 wt %), but they also contain significant amounts of organic compounds.^25,53−55^ Strong updrafts can occasionally transport biogenic organics and their oxidation products into the UTLS, where they can partition into highly acidic aerosol particles and undergo reactions similar to those described in this work. This chemistry could also be relevant in the lower atmosphere, especially in areas characterized by high emissions of SO_2_ (e.g., from burning high-sulfur fuels) and high photochemical activity. Field studies have reported highly acidic aerosols (with negative pH values) in Southeastern Asia, the eastern United States, China, and many other locations.^56−59^ Under these conditions, certain organic compounds, such as *cis*-pinonaldehyde can transform into light-absorbing products.

As sulfuric acid clouds occur on other planets, most famously on Venus,^60^ related chemical processes could occur to aldehydes and carboxylic acids should they exist in the Venusian atmosphere. While the presence of organic molecules on Venus is a matter of hot debate, nucleic acids have been shown to withstand the harsh conditions of the upper Venusian atmosphere.^61^ Furthermore, simple organic molecules can be produced in the sulfuric acid aerosol droplets from photochemically generated formaldehyde, and give rise to larger molecular weight species capable of absorbing visible radiation.^62^

The findings from this work further our understanding of the chemical reactions between sulfuric acid and organic compounds. Additional work should be done with other biogenic and anthropogenic SOA to identify whether acidic aging is a large driver in these systems. We also suggest that this chemistry should be examined at lower temperatures characteristic of UTLS as well as higher temperatures characteristic of the Venusian atmosphere because the temperature dramatically affects the rates of chemical processes and phase states of aerosol particles.^63^

## Supporting Information Available

Description of synthesis of pinonaldehyde, additional information on conditions of aging experiments, kinetic analysis, HRMS analysis and NMR analysis, and a compilation of NMR spectra of synthesized reactants and separated products of aging (PDF)
