# Supplementary material for: Formation of Chromophores from cis-Pinonaldehyde Aged in Highly Acidic Conditions
Source: J Am Chem Soc. 2024 Apr 19;146(17):11702–10. doi: 10.1021/jacs.3c14177 (PMC11066867; doi:10.1021/jacs.3c14177)
Supplement: Supplementary file 1 — ja3c14177_si_001.pdf [file ja3c14177_si_001.pdf]

Formation of chromophores from *cis*-pinonaldehyde aged in highly acidic conditions

Cynthia Wong, Jessica E. Pazienza, Scott D. Rychnovsky, Sergey A. Nizkorodov

Department of Chemistry, University of California, Irvine

Irvine, California 92697, United States

Supporting Information

Table of Contents

Additional Experimental Details 2

Synthesis of Cis-Pinonaldehyde (3): 3

Acid aging experimental conditions 5

Table S1. Solution Acidity in Aging Experiments 5

Ultra-Performance Liquid Chromatography Data Analysis 6

Kinetic Analysis 8

Large-Scale Procedure for Aging Under Acidic Conditions for NMR Analysis 9

Summary of NMR analysis 10

Key NMR Data and Structural Analysis 11

Compound 4 11

Compound 5 12

Compound 6 13

Mechanism of the Formation of Homoterpenyl Methyl Ketone 14

Mechanism of the Formation of Chromophores 5 and 6 15

Chromophore Confirmation 15

UPLC chromatograms 15

Absorption Spectra of Separated Reaction Products 17

Synthesis of 1-(3-isopropylcyclopentyl)ethan-1-one S20 via hydrogenation of 6 18

Structural analysis of S20 19

References 20

NMR Spectra 22

Additional Experimental Details

All purchased chemicals were used without further purification unless otherwise noted. The following reagents were acquired from commercial sources: *cis*-pinonic acid (Sigma Aldrich, 98%), sulfuric acid (Fisher, 96%), (–)-α-pinene (Sigma Aldrich), and dimethylsulfide (DMS) (Sigma Aldrich). $CDCl_3$ was purchased from Cambridge Isotope Laboratories. Chemical shifts (δ) were referenced to the residual solvent peak. The ^{1}H NMR spectra were recorded at 600 MHz using a Bruker AVANCE600 (cryoprobe) NMR. The ^{13}C NMR spectra were recorded at 151 MHz on the Bruker AVANCE600 NMR. All NMR spectra were taken at 25 °C. Chemical shifts (δ) are reported in parts per million (ppm) and referenced to residual solvent peak at 7.26 ppm (^{1}H) or 77.16 ppm (^{13}C) for deuterated chloroform ($CDCl_3$). The ^{1}H NMR spectral data are presented as follows: chemical shift, multiplicity (s = singlet, d = doublet, t = triplet, q = quartet, m = multiplet, dd = doublet of doublets, ddd = doublet of doublet of doublets, dddd = doublet of doublet of doublet of doublets, coupling constant(s) in hertz (Hz), and integration.

Unless otherwise stated, synthetic reactions were carried out under an atmosphere of argon. All commercially available reagents were used as received unless stated otherwise. Solvents were purchased as ACS grade or better and as HPLC-grade and passed through a solvent purification system equipped with activated alumina columns prior to use. Thin layer chromatography (TLC) was carried out using glass plates coated with a 250 μm layer of 60 Å silica gel. TLC plates were visualized with a UV lamp at 254 nm, or by staining with $KMnO_4$ stain. Liquid chromatography was performed using forced flow (flash chromatography) with an automated purification system on prepacked silica gel (SiO_2) columns unless otherwise stated.

Synthesis of Cis-Pinonaldehyde (3):

Cis-pinonaldehyde was synthesized based on the following published procedures.[1,2]

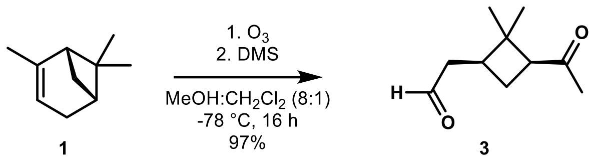

A round bottom flask containing a stirred solution of (–)-α-pinene (**1**) (0.5 mL, 3.16 mmol, 1 equiv) dissolved in a solution of MeOH (28 mL) and CH_2Cl_2 (3.5 mL) was cooled to –78 °C. Ozone (O_3) was bubbled through the solution via a commercial ozone generator until a blue color persisted. The ozone generator was turned off and O_2 bubbled through the solution until the blue color faded. While stirring at –78 °C, DMS (4.2 mL, 56.9 mmol, 18 equiv) was added dropwise. The solution was stirred for 16 h while gradually warming to rt as the dry ice/acetone bath evaporated. The reaction mixture was diluted with water, extracted with CH_2Cl_2 (4 x 30 mL), washed with brine (1 x 60 mL), dried over Na_2SO_4, filtered, and concentrated under reduced pressure to afford crude pinonaldehyde as a clear, colorless oil. The mixture was purified via flash chromatography (15% EtOAc/Hex isocratic) to afford **3** as a clear, colorless oil (417 mg, 97%) containing trace amounts of EtOAc. Product was too volatile to be dried under high vacuum.

^{1}H NMR (500 MHz, $CDCl_3$): δ 9.63 (s, 1H), 2.83 (dd, *J* = 10.0, 7.8 Hz, 1H), 2.57 – 2.20 (m, 3H), 1.94 (s, 3H), 1.85 (ddd, *J* = 19.1, 9.2, 2.5 Hz, 2H), 1.24 (s, 3H), 0.74 (s, 3H).

^{13}C{^{1}H} NMR (126 MHz, $CDCl_3$): δ 207.3, 201.3, 54.2, 45.0, 43.2, 35.7, 30.2, 30.1, 22.7, 17.5.

HRMS (Orbitrap, m/Δm = 1.4 × 10^5**)** *m/z*: Calcd for $C_{10}H_{17}O_2^+$ $[M+H]^+$ 169.1223; Found 169.1223

Acid aging experimental conditions

Table S1. Solution Acidity in Aging Experiments

		E-AIM Output*				
Estimated Concentration** of H_2SO_4	pH Meter Reading	$[HSO_4^-]$ (mol/kg)	$[SO_4^-]$ (mol/kg)	$[H^+]$ (mol/kg)	Activity Coefficient of H^+	Effective pH = -log$[H^+]$
0 M (Control)	4.3	-	-	-	-	-
0.52 mM	2.7	0.0000386	0.000481	0.00100	0.96	3.00
90 mM	1.1	0.0654	0.0249	0.115	0.76	0.94
1.0 M	-	0.754	0.249	1.25	0.78	-0.01
1.8 M	-	1.26	0.508	2.28	0.92	-0.36
3.2 M	-	2.15	1.01	4.17	1.4	-0.62
5.6 M	-	3.96	1.67	7.30	3.7	-0.86
10 M	-	8.10	1.93	12.0	20	-1.08

* The extended aerosol inorganic model I (E-AIM) was utilized to estimate the molalities and activities of ions present in the sulfuric acid solutions.[3–5] For consistency, we will use the effective pH in the last column to refer to experimental conditions used in this work

** All solutions used in this work used deionized water as solvent.

Ultra-Performance Liquid Chromatography Data Analysis

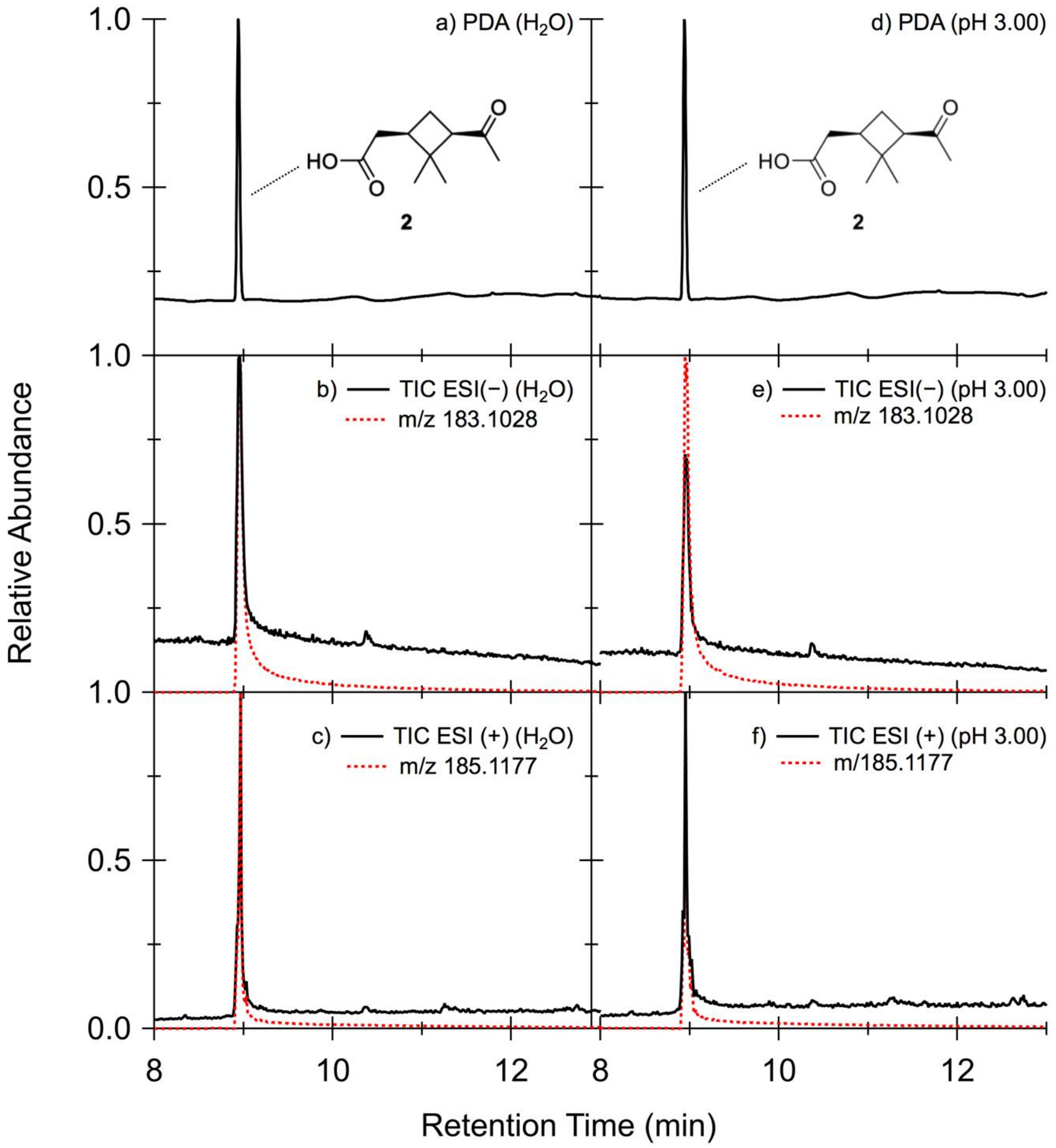

Figure S1. UPLC chromatograms of *cis*-pinonic acid (**2**) aged in H_2O and 0.52 mM H_2SO_4 (pH 3.00) for 2 days in ESI(–) mode (b, e), ESI(+) mode (c, f), and the corresponding PDA (a, d). Overlaid on the negative ion mode and positive ion mode TIC in red is the SIC for *m/z* 183.1028 and *m/z* 185.1177. The PDA chromatograms were shifted 0.06 minutes to account for the time delay between the two detectors. All chromatograms were normalized based on the maximum peak intensity of their respective dataset.

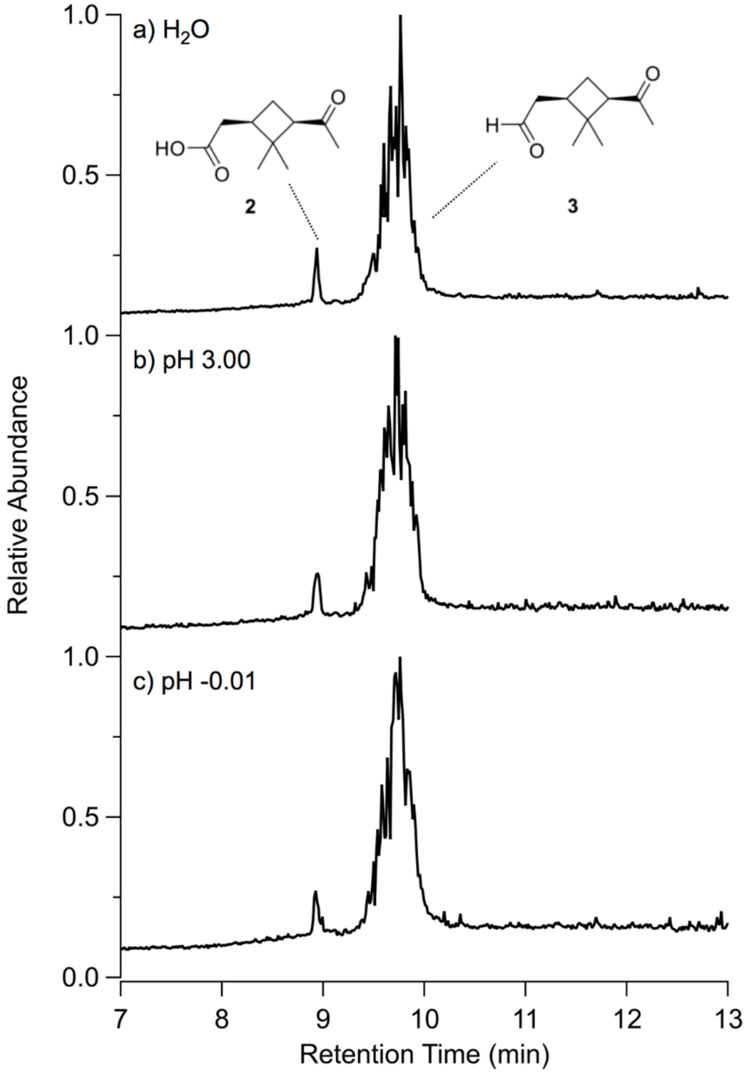

Figure S2. Positive ion mode TIC for *cis*-pinonaldehyde (3) aged in (a) H_2O, (b) 0.52 mM H_2SO_4 (pH 3.00), and (c) 1.0 M H_2SO_4 (pH -0.01).

Kinetic Analysis

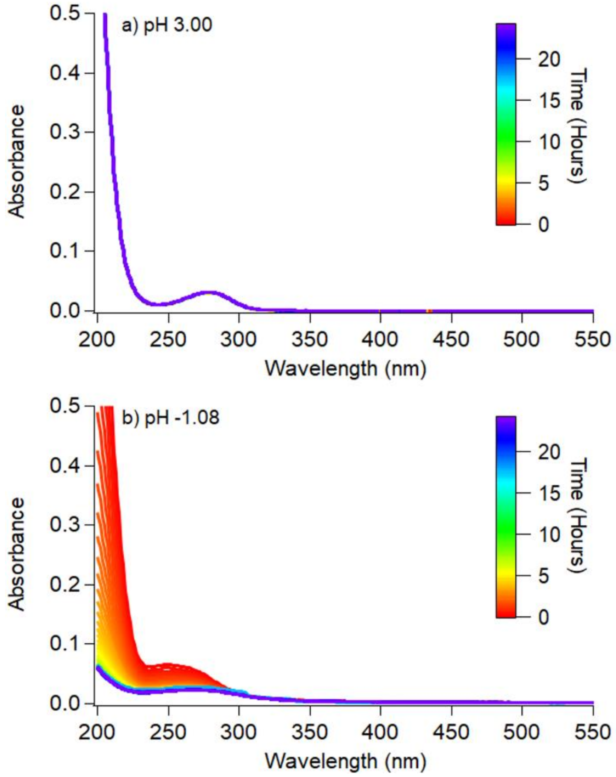

Figure S3. Absorption spectra of *cis*-pinonic acid (2) aged in (a) 5.2 x 10^{-4} M (pH 3.00), and (b) 10 M (pH −1.08) solutions of H_2SO_4 recorded every 15 min for 24 h.

Figure S3 shows the absorption spectra of *cis*-pinonic acid (2) aged in (a) 5.2 x 10^{-4} M (pH 3.00), and (b) 10 M (pH −1.08) solutions of H_2SO_4 as a function of time. The spectra of *cis*-pinonic acid in moderately acidic solutions (pH 3.00) are consistent with the presence of non-interacting carboxyl and carbonyl groups. The small peak at 285 nm corresponds to the weak n → π* transition in the carbonyl group of *cis*-pinonic acid (2), and the larger peak below 200 nm corresponds to π → π* transitions. As seen in Figure S3a, there is no change in either the n → π* or π → π* transition as function of time *cis*-pinonic acid (2) confirming that *cis*-pinonic acid (2) is stable under these conditions. In Figure S3b, there is also the presence of the weak n → π* transition (~262 nm) and π → π* transition (< 200 nm) in the *cis*-pinonic acid (2) sample aged in 10 M (pH −1.08) H_2SO_4.

However, the peaks that correspond to those transitions decrease over time, indicating a structural change (e.g., tautomerization). Previous studies have shown that ketones can undergo acid-catalyzed aldol condensation, however, the aldol condensation product resulting from *cis*-pinonic acid (**2**) , $C_{20}H_{30}O_5$, is absent from the mass spectra.[6,7] The evidence presented in the TIC (Figure S1) and UV-Vis spectra (Figure S3b) suggest that there is a structural change (e.g., tautomerization) in *cis*-pinonic acid (**2**) rather than condensation reaction. In the first three hours, the reaction behaves as a first order reaction with respect to the decay of *cis*-pinonic acid (**2**), resulting in a rate constant of 6.33×10^{-5} h^{-1}. Arcus and Bennett (1955), reported a rate constant of 9.9×10^{-2} h^{-1},[8] however, their conversion of *cis*-pinonic acid (**2**) to homoterpenyl methyl ketone (**4**) was done in monochloroacetic acid at 100°, while the reaction in this paper was done with sulfuric acid at room temperature and a lower concentration of starting material.[8] This suggests that the isomerization rate constant strongly depends on temperature, which is an important consideration given the wide range of ambient temperatures in the atmosphere.

Large-Scale Procedure for Aging Under Acidic Conditions for NMR Analysis

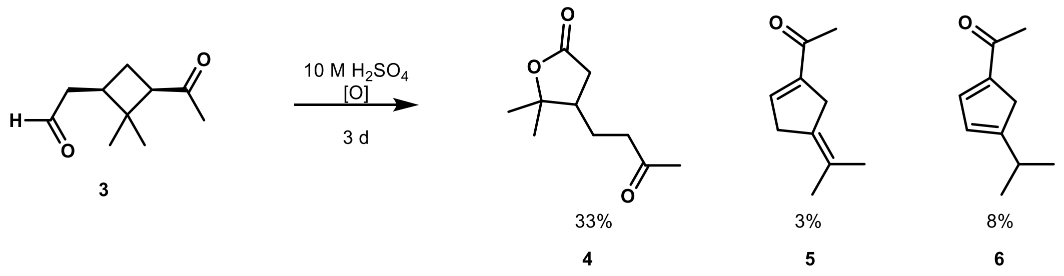

A solution of **3** (8.77 g, 52.1 mmol) in ~500 mL of 10 M H_2SO_4 (pH –1.08), resulting in a mass concentration of ~17 mg/mL, was prepared. The solution was aged for 3 days in an open atmosphere. The crude solution was then poured over ice and diluted with H_2O. Solid $NaHCO_3$ was added portion wise while stirring vigorously and monitoring pH. When the pH was

approximately 2, the solution was partitioned with CH_2Cl_2, extracted with CH_2Cl_2, and washed with a saturated aqueous solution of $NaHCO_3$ and a saturated solution of NaCl. The organic layers were dried over Na_2SO_4 and concentrated in vacuo to afford a crude black oil. The residue was purified via flash chromatography (0-100% EtOAc/Hex) to afford **4** (3.14 g, 33%), **5** (233 mg, 3.0%) as an inseparable mixture with another constitutional isomer, and **6** (622 mg, 8.0%).

Summary of NMR analysis

Table S2. Tabulations of the ^{1}H and ^{13}C NMR data for compounds **4, 5,** and **6.**

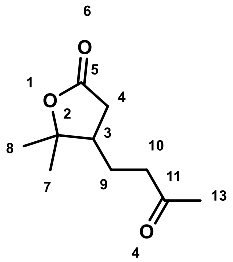

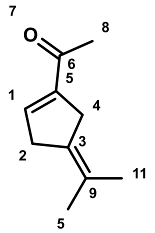

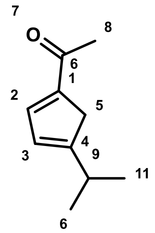

Pos.	4 δH, mult	4 δc	5 δH, mult	5 δc	6 δH, mult	6 δc
1			7.16 (s, 1H)	140.9		145.0
2		86.7	2.63 (d, 6.5, 2H)	29.3	7.24 (s, 1H)	143.9
3	2.17 (tdd, 11.1, 7.8, 3.8, 1H)	45.2		133.1	6.26 – 6.16 (m, 1H)	125.0
4	2.55 (dd, 17.0, 7.9, 1H)<br>2.24 (dd, 17.0, 11.7, 1H)	34.8	2.53 (d, 4.4, 2H)	27.8		166.1
5		175.3		146.3	3.22 (d, 1.5, 2H)	40.2
6				197.1		193.7
7	1.24 (s, 3H)	21.9				
8	1.42 (s, 3H)	27.4	2.33 (s, 3H)	26.7	2.33 – 2.30 (m, 3H)	26.0
9	1.77 (dddd, 13.5, 9.2, 6.6, 4.0, 1H)<br>1.51 (dddd, 13.9, 10.7, 8.7, 5.7, 1H)	23.3		140.5	2.72 (dtd, 13.7, 6.9, 1.2, 1H)	30.3
10	2.50 – 2.38 (m, 2H)	41.9	1.74 (s, 3H)	22.0	1.14 (ddd, 6.9, 2.0, 0.9, 8H).	22.8
11		207.3	1.87 (s, 3H)	21.5	1.14 (ddd, 6.9, 2.0, 0.9, 8H).	22.8
12						
13	2.14 (s, 3H)	30.1				

Key NMR Data and Structural Analysis

Compound 4

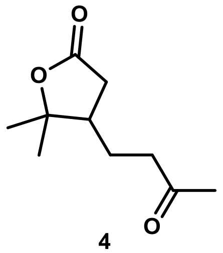

4

5,5-dimethyl-4-(3-oxobutyl)dihydrofuran-2(3H)-one (**4**)

^{1}H NMR (600 MHz, $CDCl_3$) δ 2.55 (dd, *J* = 17.0, 7.9 Hz, 1H), 2.50 – 2.38 (m, 2H), 2.24 (dd, *J* = 17.0, 11.7 Hz, 1H), 2.17 (tdd, *J* = 11.1, 7.8, 3.8 Hz, 1H), 2.14 (s, 3H), 1.77 (dddd, *J* = 13.5, 9.2, 6.6, 4.0 Hz, 1H), 1.51 (dddd, *J* = 13.9, 10.7, 8.7, 5.7 Hz, 1H), 1.42 (s, 3H), 1.24 (s, 3H).

^{13}C NMR (151 MHz, $CDCl_3$) δ 207.3, 175.3, 86.7, 45.2, 41.9, 34.8, 30.1, 27.4, 23.3, 21.9.

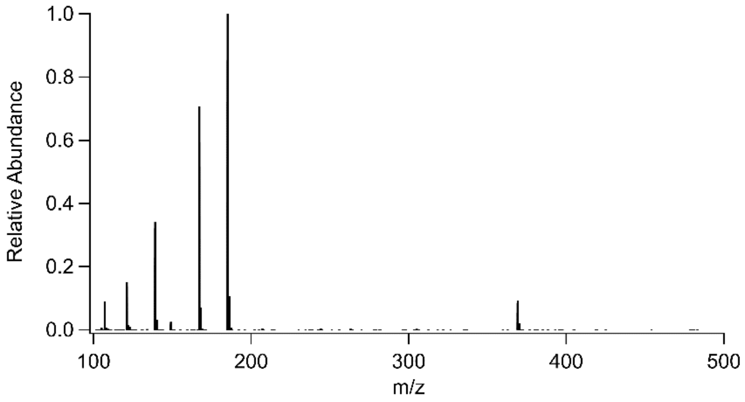

HRMS (+): The compound was identified to be *m/z* 185.1177 with a molecular formula of $[C_{10}H_{16}O_3{+}H]^{+\cdot}$ Other peaks are the fragments of *m/z* 185.1177.

Compound 5

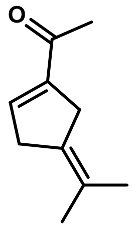

5

1-(4-(propan-2-ylidene)cyclopent-1-en-1-yl)ethan-1-one (**5**)

^{1}H NMR (600 MHz, $CDCl_3$) δ 7.16 (s, 1H), 2.63 (d, *J* = 6.5 Hz, 2H), 2.53 (d, *J* = 4.4 Hz, 2H), 2.33 (s, 3H), 1.87 (s, 3H), 1.74 (s, 3H).

^{13}C NMR (151 MHz, $CDCl_3$) δ 197.11, 146.34, 140.85, 140.49, 132.06, 29.46, 27.79, 26.65, 21.98, 21.45.

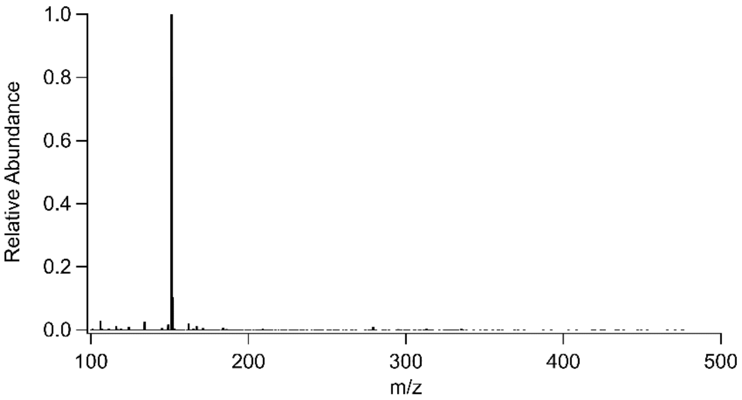

HRMS (+): The compound was identified to be *m/z* 151.1118 with a molecular formula of $[C_{10}H_{14}O+H]^+$

Compound 6

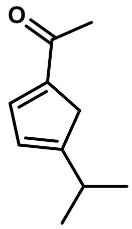

6

1-(4-isopropylcyclopenta-1,3-dien-1-yl)ethan-1-one (**6**)

^{1}H NMR (600 MHz, $CDCl_3$) δ 7.24 (s, 1H), 6.26 – 6.16 (m, 1H), 3.22 (d, *J* = 1.5 Hz, 2H), 2.72 (dtd, *J* = 13.7, 6.9, 1.2), 2.33 – 2.30 (m, 3H), 1.14 (ddd, *J* = 6.9, 2.0, 0.9 Hz, 6H).

^{13}C NMR (151 MHz, $CDCl_3$) δ 193.71, 166.11, 144.95, 143.94, 125.00, 40.21, 30.34, 25.97, 22.75.

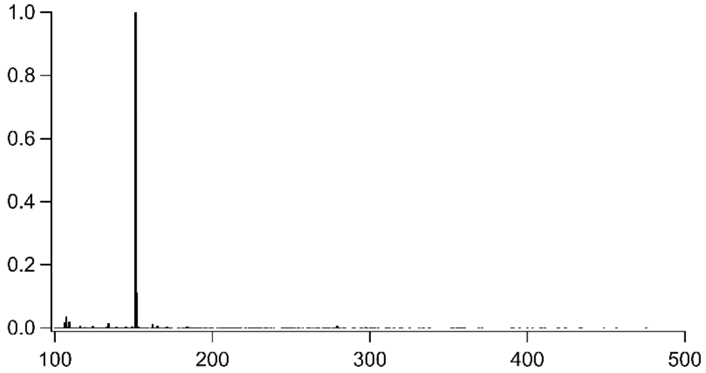

HRMS (+): The compound was identified to be *m/z* 151.1118 with a molecular formula of $[C_{10}H_{14}O+H]^+$

Mechanism of the Formation of Homoterpenyl Methyl Ketone

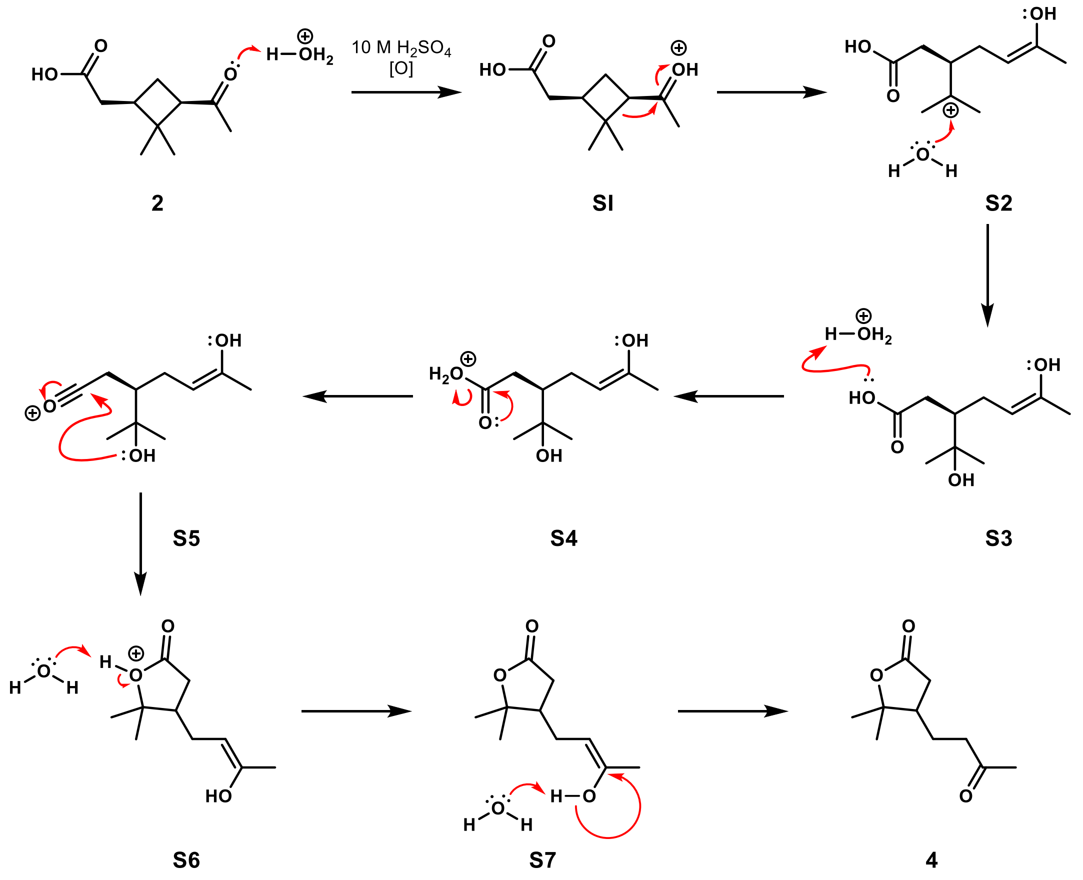

Scheme S1. Formation of homoterpenyl methyl ketone (**4**) from *cis*-pinonic acid (**2**) aged in H_2SO_4, adapted from Arcus and Bennett (1955)[8]. Carboxylic acid **2** undergoes an acid-catalyzed ring opening to afford enol **S2** followed by tandem elimination of water and cyclization of **S5** to form the γ-lactone **4**.

Mechanism of the Formation of Chromophores 5 and 6

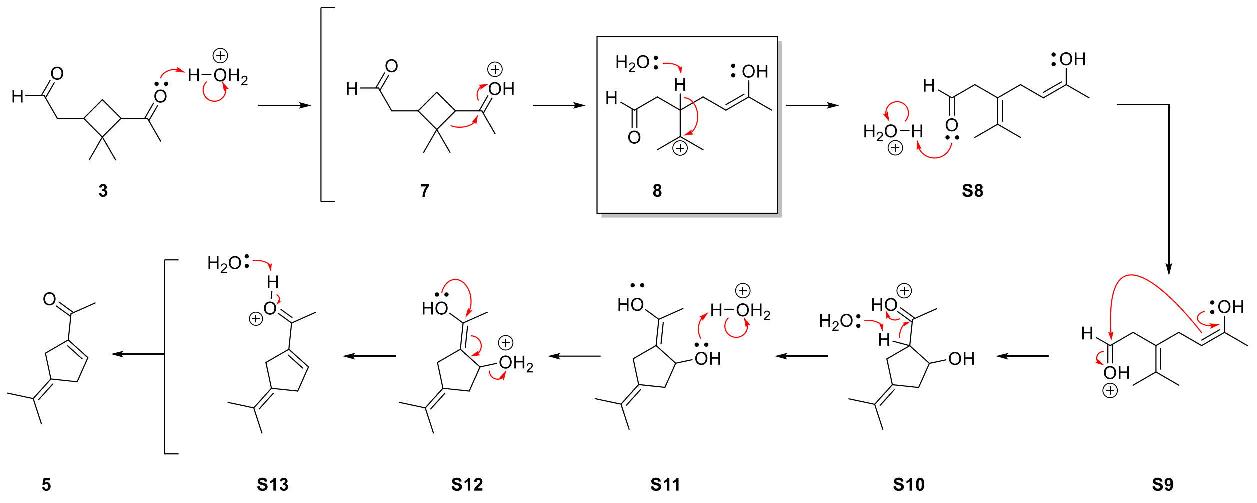

Scheme S2. Mechanism of formation of chromophore **5**. Common intermediate **8** is boxed.

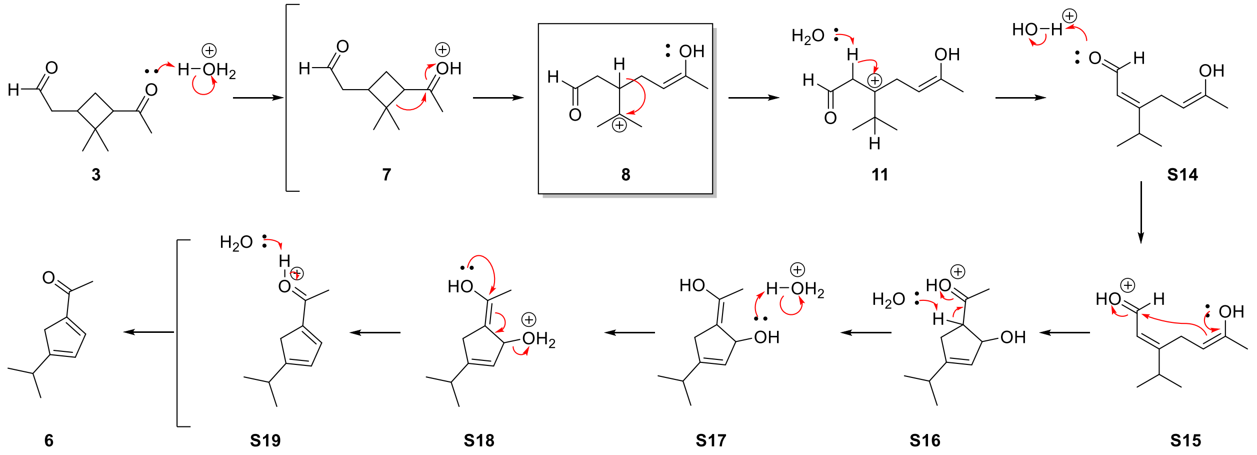

Scheme S3. Mechanism of formation of chromophore **6**. Common intermediate **8** is boxed.

Chromophore Confirmation

UPLC chromatograms

To confirm that purified samples were the chromophores of interest, purified extracts were analyzed using UPLC-PDA-HRMS and then compared the peaks and elution times of Figure 3. Figure S4 shows the UPLC chromatograms of the separated samples, Compound **5** (blue trace) and 6 (orange trace).

In the Compound **5** isolated sample, the TIC (Figure S4a) contains 2 peaks, one at 11.03 min and another at 12.17 min. Both peaks absorb as seen in Figure S4b, however, only the peak that eluted out at 12.17 min has the matches the TIC retention time in *cis*-pinonaldehyde (**3**) solution (Figure 3) and has *m/z* 151.1117 ($C_{10}H_{15}O^+$). This peak at 11.03 min is likely an impurity due to the purification process as this peak is not evident in Figure 3 and has a mass of *m/z* 149.0961 ($C_{10}H_{13}O^+$). In Compound **6**, the TIC (Figure S4a) also contains 2 peaks, one at 11.79 min and another at 12.00 min. Both peaks absorb and have *m/z* 151.1118 as noted by Figure S4b and Figure S4c.

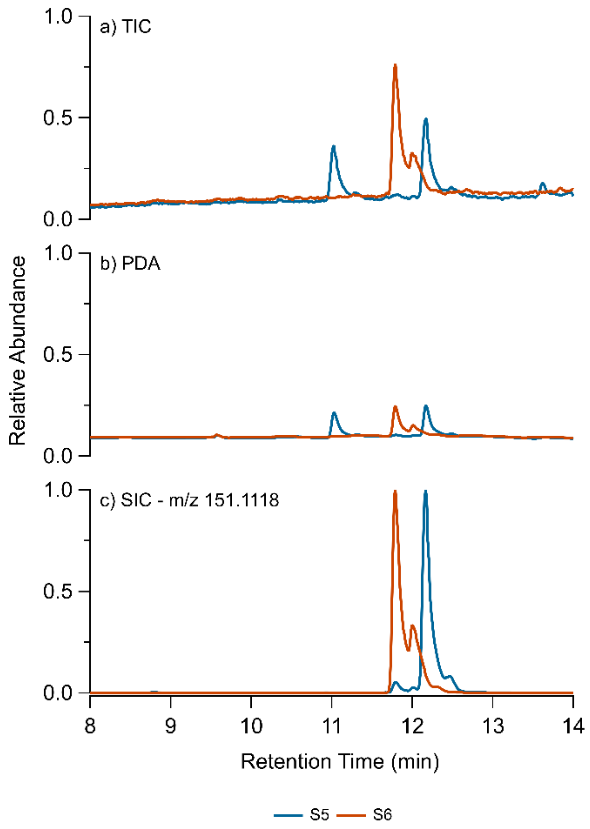

Figure S4. UPLC chromatograms of 5 and 6 isolated extracts in positive ion mode for TIC (a), PDA (b), and SIC (*m/z* 151.1117) (c). The PDA chromatogram was shifted ~0.06 minutes to account for the time delay between the two detectors.

In comparison to the elution time in Figure 3 in the main text, it appears that Compound **5** corresponds to peak at 12.21 min while Compound **6** corresponds to the peak at 11.85 and 12.07 min. Note that there is a ~0.05 min delay time between the purified samples (Figure S4) and the unpurified sample (Figure 3), which is likely due to instrument fluctuations as these samples were taken a couple of weeks apart. Additionally, the constitutional isomer that corresponds to the peak at 12.07 min could not be further purified and the structure determination is limited as there were only trace amounts in the scaled-up reaction so compound will have limited discussion in the following sections.

Absorption Spectra of Separated Reaction Products

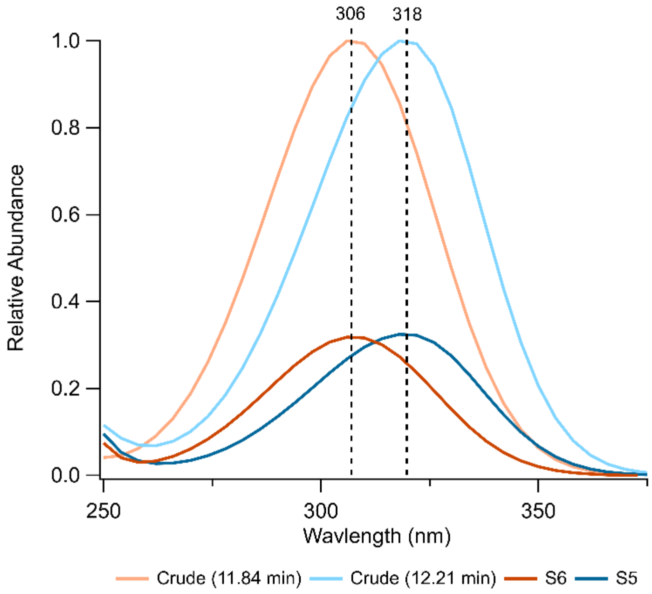

Figure S5. Absorption spectra from PDA from the crude and isolated samples

Additional comparisons for the purified and crude samples are shown in Figure S5, which highlights the respective PDA absorption spectra. The spectra for compound **5** and peak at 12.21 min in Figure 3 absorb the same max wavelength of 318 nm, while the compound **6** sample likely corresponds to the peak at 11.84 min in Figure 3 because each peak absorbs at 306 nm. Note that the maximum absorption wavelength differs from Figure 4b, which is likely caused by a shift in

the acid base equilibria as the samples were diluted with water–acetonitrile eluent used in UPLC.[9] This suggests that absorption spectra of these compounds are pH dependent, which is characteristic of other atmospheric compounds like nitrophenols, imidazole-2-carboxaldehyde and pyruvic acid.[10–14] Furthermore, due to this pH dependence it is unclear if these compounds account for the absorption at 415 nm and 500 nm, as depicted in Figure 4, which may suggests the presence of other chromophores that could be generated during the aging *cis*-pinonaldehyde aged in 10 M (pH −1.08) H_2SO_4.

Synthesis of 1-(3-isopropylcyclopentyl)ethan-1-one S20 via hydrogenation of 6

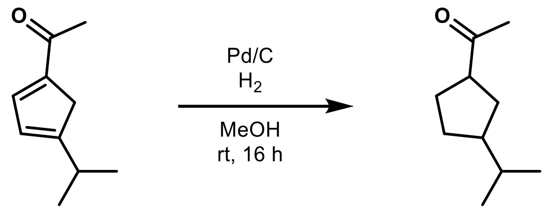

6 **S20**

A sample of **6** was azeotroped in MeOH prior to subjecting to the reaction conditions.

In a two-neck round bottom flask under argon atmosphere was prepared a solution of Pd/C in MeOH (sparged for 1 h) and sparged for an additional 30 min. **6** was added as a solution in MeOH (sparged for 30 min). The solution was then placed under a hydrogen atmosphere (hydrogen balloon; sparged for 2 h) and stirred for 16 h at room temperature. The solution was concentrated in vacuo to afford **S20** as a 1:1 mixture of diastereomers. The sample was used crude for analysis.

^{1}H NMR (600 MHz, $CDCl_3$): δ 2.98 – 2.81 (m, 1H), 2.14 (s, 3H), 2.03 – 1.96 (m, 1H), 1.94 – 1.88 (m, 1H), 1.86 – 1.80 (m, 1H), 1.79 – 1.71 (m, 1H), 1.71 – 1.61 (m, 1H), 1.58 – 1.50 (m, 1H), 1.42 – 1.33 (m, 1H), 1.32 – 1.25 (m, 1H), 0.92 – 0.85 (m, 6H).

^{13}C{^{1}H} NMR (151 MHz, $CDCl_3$): δ 211.6, 211.5, 52.4, 51.7, 48.7, 47.3, 34.4, 33.53, 33.48, 32.6, 31.7, 30.2, 29.1, 29.0, 28.8, 27.6, 21.8, 21.72, 21.70, 21.5.

Structural analysis of S20

1D and 2D NMR experiments of unknown compound **6** indicated that the structure was one of two possible options – compound **S21** or the structure we have assigned as compound **6** – which differ in the substitution pattern of the acyl and isopropyl moieties, either 1,2- or 1,3- around the ring. **S22**, the hydrogenation product of **S21**, is known in the literature (Figure S6) and reported and characterized by Hoveyda and coworkers.[15] They observe a ^{1}H shift of the C1 methine at 3.10–3.06 for the syn-diastereomer and 2.63 for the anti-diastereomer of **S22**. We rationalized that if we observed these same signals following hydrogenation of compound **6**, our unknown would follow a 1,2-substitution pattern. If we observed differing signals at the C1 methine, we would have the 1,3-substitution pattern adduct.

Hydrogenation of **6** afforded **S20** as a 1:1 mixture of diastereomers (refer to 20 peaks observed in the ^{13}C NMR for a molecular formula of $C_{10}H_{18}O$). Indicative peaks include the methyl ketone COC**H$_3$** at 2.14 ppm and the isopropyl group CH(C**H$_3$**) at 0.92–0.85 ppm. Notably, we observe the C1 methine at 2.85–2.93, with both the syn- and anti-diastereomers overlapping. As this shift differs from those reported by the Hoveyda group, we concluded that unknown **6** demonstrates a 1,3-substitution pattern around the ring.

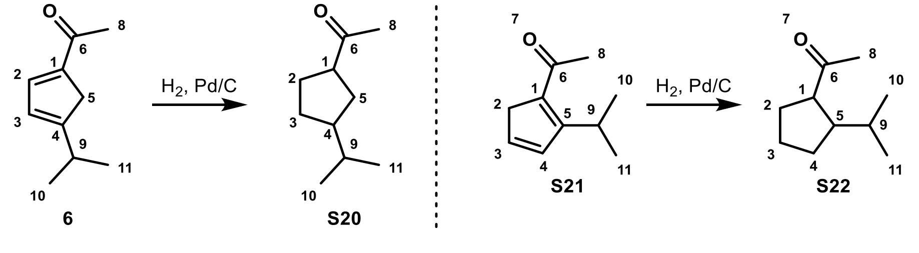

Figure S6. Possible hydrogenation products of unknown **6.**

References

(1) Nguyen, T. B.; Laskin, A.; Laskin, J.; Nizkorodov, S. A. Brown Carbon Formation from Ketoaldehydes of Biogenic Monoterpenes. *Faraday Discuss* **2013**, *165* (0), 473–494. https://doi.org/10.1039/C3FD00036B.

(2) Zhang, Y.; Apsokardu, M. J.; Kerecman, D. E.; Achtenhagen, M.; Johnston, M. V. Reaction Kinetics of Organic Aerosol Studied by Droplet Assisted Ionization: Enhanced Reactivity in Droplets Relative to Bulk Solution. *J Am Soc Mass Spectrom* **2021**, *32* (1), 46–54. https://doi.org/10.1021/jasms.0c00057.

(3) Carslaw, K. S.; Clegg, S. L.; Brimblecombe, P. A Thermodynamic Model of the System HCl-HNO3-H2SO4-H2O, Including Solubilities of HBr, from <200 to 328 K. *Journal of Physical Chemistry* **1995**, *99* (29), 11557–11574. https://doi.org/10.1021/J100029A039.

(4) Massucci, M.; Clegg, S. L.; Brimblecombe, P. Equilibrium Partial Pressures, Thermodynamic Properties of Aqueous and Solid Phases, and Cl2 Production from Aqueous HCl and HNO3 and Their Mixtures. *Journal of Physical Chemistry A* **1999**, *103* (21), 4209–4226. https://doi.org/10.1021/JP9847179.

(5) Clegg, S. L.; Brimblecombe, P.; Wexler, A. S. *Extended AIM Aerosol Thermodynamics Model*. http://www.aim.env.uea.ac.uk/aim/aim.php.

(6) Nozière, B.; Esteve, W. Organic Reactions Increasing the Absorption Index of Atmospheric Sulfuric Acid Aerosols. *Geophys Res Lett* **2005**, *32* (3), 1–5. https://doi.org/10.1029/2004GL021942.

(7) Nozière, B.; Esteve, W. Light-Absorbing Aldol Condensation Products in Acidic Aerosols: Spectra, Kinetics, and Contribution to the Absorption Index. *Atmos Environ* **2007**, *41* (6), 1150–1163. https://doi.org/10.1016/J.ATMOSENV.2006.10.001.

(8) Arcus, C. L.; Bennett, G. J. The Mechanism of the Rearrangement of Pinonic Acid into Homoterpenyl Methyl Ketone. *Journal of the Chemical Society (Resumed)* **1955**, *28* (0), 2627–2632. https://doi.org/10.1039/JR9550002627.

(9) Wong, C.; Liu, S.; Nizkorodov, S. A. Highly Acidic Conditions Drastically Alter the Chemical Composition and Absorption Coefficient of α-Pinene Secondary Organic Aerosol. *ACS Earth Space Chem* **2022**, *6* (12), 2983–2994. https://doi.org/doi/10.1021/acsearthspacechem.2c00249.

(10) Vione, D.; Maurino, V.; Minero, C.; Duncianu, M.; Olariu, R. I.; Arsene, C.; Sarakha, M.; Mailhot, G. Assessing the Transformation Kinetics of 2- and 4-Nitrophenol in the Atmospheric Aqueous Phase. Implications for the Distribution of Both Nitroisomers in the Atmosphere. *Atmos Environ* **2009**, *43* (14), 2321–2327. https://doi.org/10.1016/J.ATMOSENV.2009.01.025.

(11) Barsotti, F.; Bartels-Rausch, T.; De Laurentiis, E.; Ammann, M.; Brigante, M.; Mailhot, G.; Maurino, V.; Minero, C.; Vione, D. Photochemical Formation of Nitrite and Nitrous Acid (HONO) upon Irradiation of Nitrophenols in Aqueous Solution and in Viscous Secondary Organic Aerosol Proxy. *Environ Sci Technol* **2017**, *51* (13), 7486–7495. https://doi.org/10.1021/acs.est.7b01397.

(12) Lee, H. J.; Aiona, P. K.; Laskin, A.; Laskin, J.; Nizkorodov, S. A. Effect of Solar Radiation on the Optical Properties and Molecular Composition of Laboratory Proxies of Atmospheric Brown Carbon. *Environ Sci Technol* **2014**, *48* (17), 10217–10226. https://doi.org/10.1021/es502515r.

(13) Ackendorf, J. M.; Ippolito, M. G.; Galloway, M. M. PH Dependence of the Imidazole-2-Carboxaldehyde Hydration Equilibrium: Implications for Atmospheric Light Absorbance. *Environ Sci Technol Lett* **2017**, *4* (12), 551–555. https://doi.org/10.1021/acs.estlett.7b00486.

(14) Tilgner, A.; Schaefer, T.; Alexander, B.; Barth, M. C.; Collett, J. L.; Fahey, K. M.; Nenes, A.; Pye, H. O. T.; Herrmann, H.; McNeill, V. F. Acidity and the Multiphase Chemistry of Atmospheric Aqueous Particles and Clouds. *Atmos Chem Phys* **2021**, *21* (17), 13483–13536. https://doi.org/10.5194/ACP-21-13483-2021.

(15) Degrado, S. J.; Mizutani, H.; Hoveyda, A. H. Efficient Cu-Catalyzed Asymmetric Conjugate Additions of Alkylzincs to Trisubstituted Cyclic Enones. *J Am Chem Soc* **2002**, *124* (45), 13362–13363. https://doi.org/10.1021/ja021081x.

NMR Spectra

The following pages contain images of NMR spectra of the compounds used in this work.

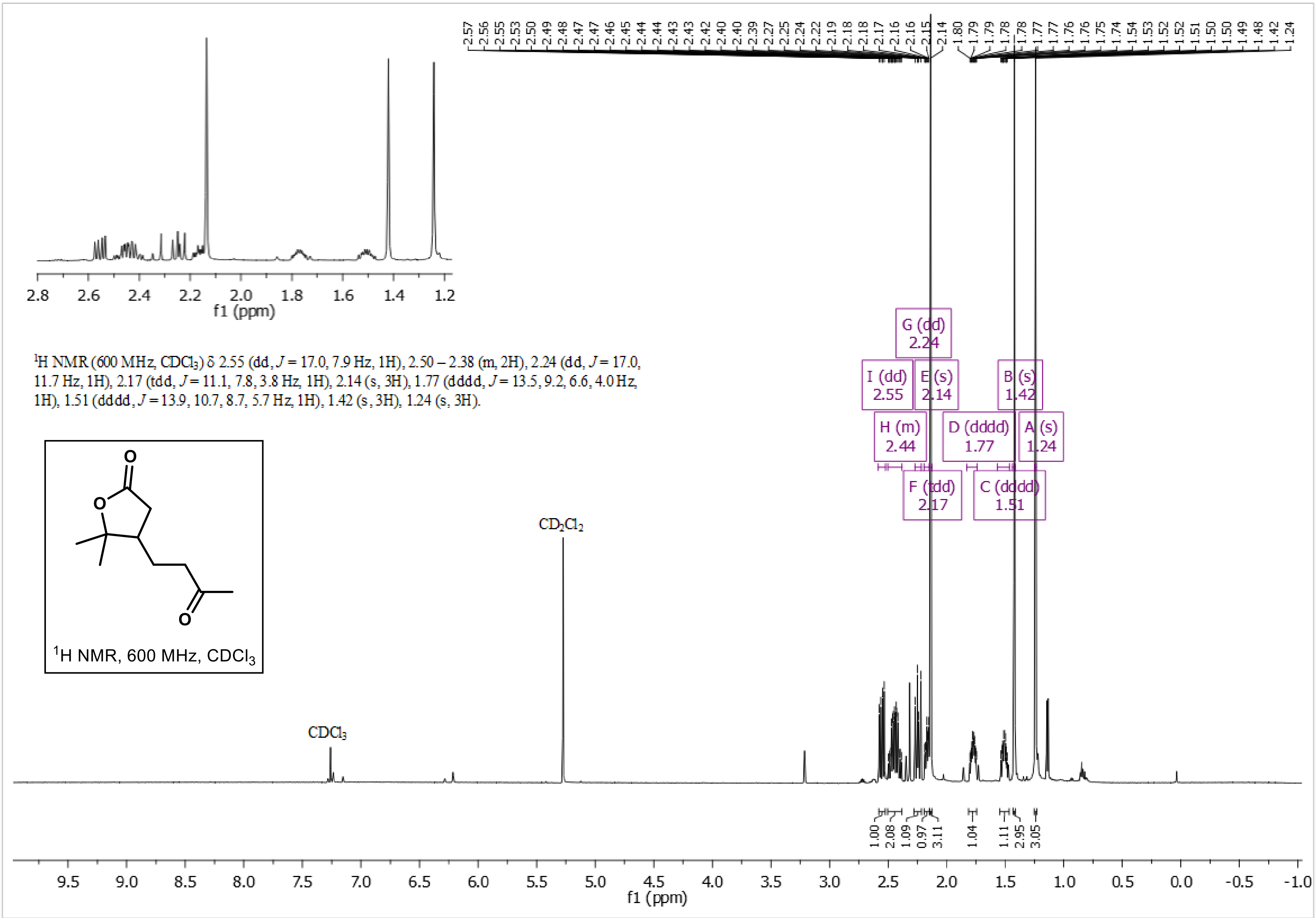
2.57 2.56 2.55 2.53 2.50 2.49 2.48 2.47 2.47 2.46 2.45 2.44 2.44 2.43 2.43 2.42 2.40 2.40 2.39 2.27 2.25 2.24 2.22 2.19 2.18 2.17 2.16 2.16 2.15 2.14 1.80 1.79 1.79 1.78 1.78 1.77 1.77 1.76 1.75 1.74 1.54 1.53 1.52 1.52 1.51 1.50 1.50 1.49 1.48 1.42 1.24
f1 (ppm)
^{1}H NMR (600 MHz, $CDCl_3$) δ 2.55 (dd, J = 17.0, 7.9 Hz, 1H), 2.50 – 2.38 (m, 2H), 2.24 (dd, J = 17.0, 11.7 Hz, 1H), 2.17 (tdd, J = 11.1, 7.8, 3.8 Hz, 1H), 2.14 (s, 3H), 1.77 (dddd, J = 13.5, 9.2, 6.6, 4.0 Hz, 1H), 1.51 (dddd, J = 13.9, 10.7, 8.7, 5.7 Hz, 1H), 1.42 (s, 3H), 1.24 (s, 3H).
G (dd) 2.24
I (dd) 2.55
E (s) 2.14
B (s) 1.42
H (m) 2.44
D (dddd) 1.77
A (s) 1.24
F (tdd) 2.17
C (dddd) 1.51
CD_2Cl_2
$CDCl_3$
^{1}H NMR, 600 MHz, $CDCl_3$
1.00 2.08 1.09 0.97 3.11 1.04 1.11 2.95 3.05
f1 (ppm)

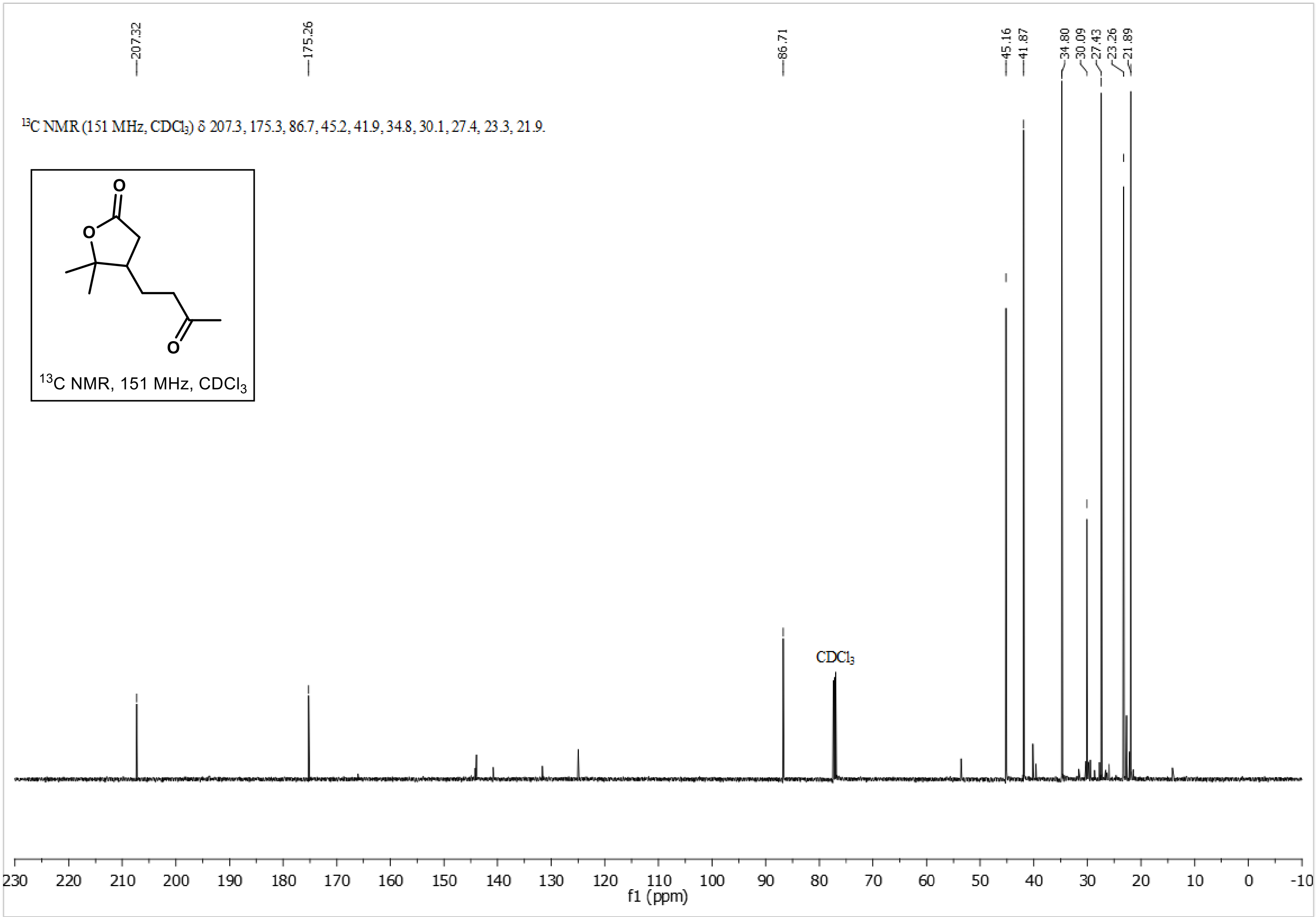
207.32
175.26
86.71
45.16
41.87
34.80
30.09
27.43
23.26
21.89
13C NMR (151 MHz, CDCl3) δ 207.3, 175.3, 86.7, 45.2, 41.9, 34.8, 30.1, 27.4, 23.3, 21.9.
O
O
O
13C NMR, 151 MHz, CDCl3
CDCl3
230 220 210 200 190 180 170 160 150 140 130 120 110 100 90 80 70 60 50 40 30 20 10 0 -10
f1 (ppm)

^{1}H NMR (600 MHz, $CDCl_3$) δ 7.16 (s, 1H), 2.63 (d, J = 6.5 Hz, 2H), 2.53 (d, J = 4.4 Hz, 2H), 2.33 (s, 3H), 1.87 (s, 3H), 1.74 (s, 3H).

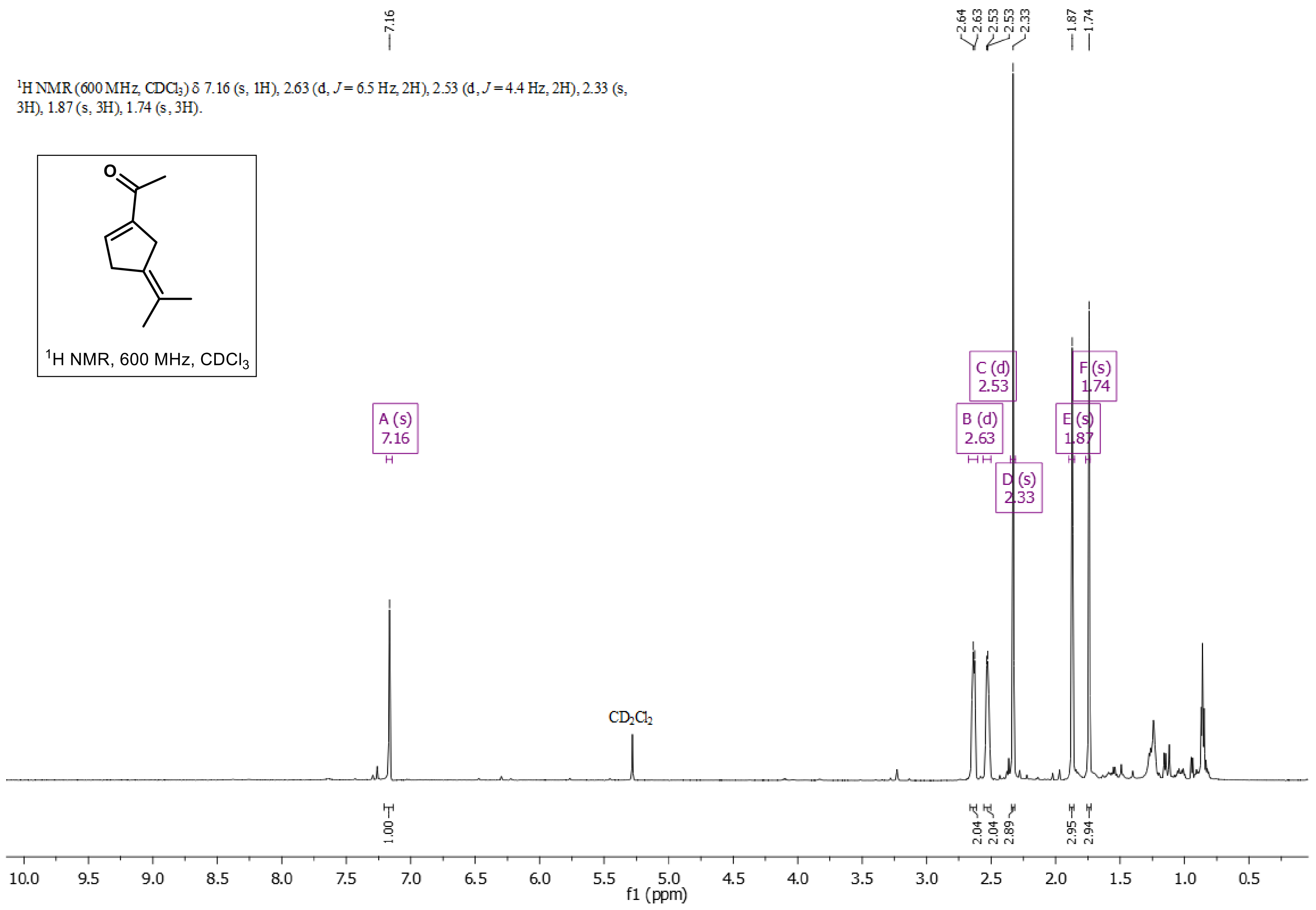

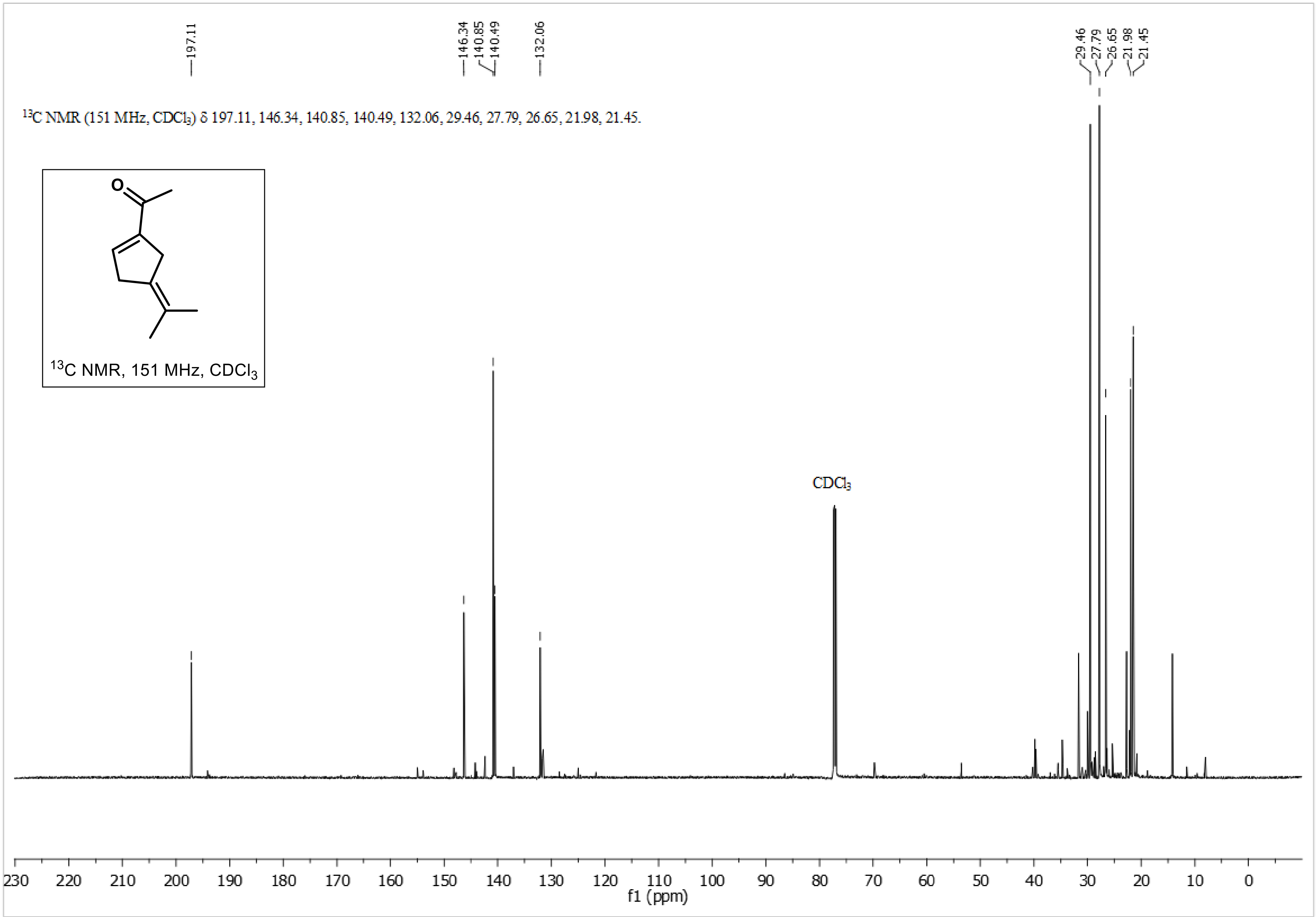
197.11
146.34
140.85
140.49
132.06
29.46
27.79
26.65
21.98
21.45
^{13}C NMR (151 MHz, $CDCl_3$) δ 197.11, 146.34, 140.85, 140.49, 132.06, 29.46, 27.79, 26.65, 21.98, 21.45.
O
^{13}C NMR, 151 MHz, $CDCl_3$
$CDCl_3$
230 220 210 200 190 180 170 160 150 140 130 120 110 100 90 80 70 60 50 40 30 20 10 0
f1 (ppm)

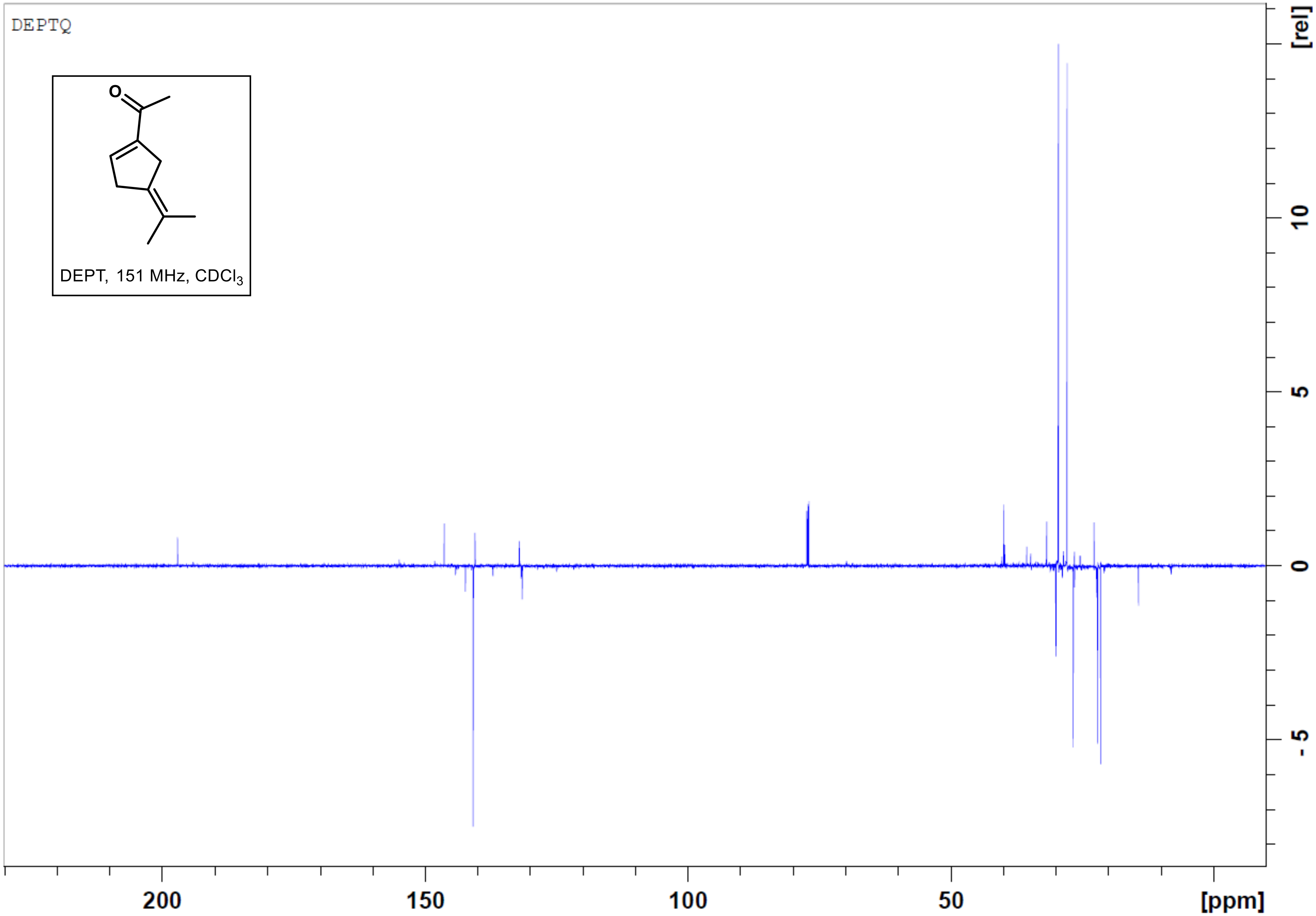

DEPTQ
O
DEPT, 151 MHz, CDCl3
[rel]
10
5
0
-5
200
150
100
50
[ppm]

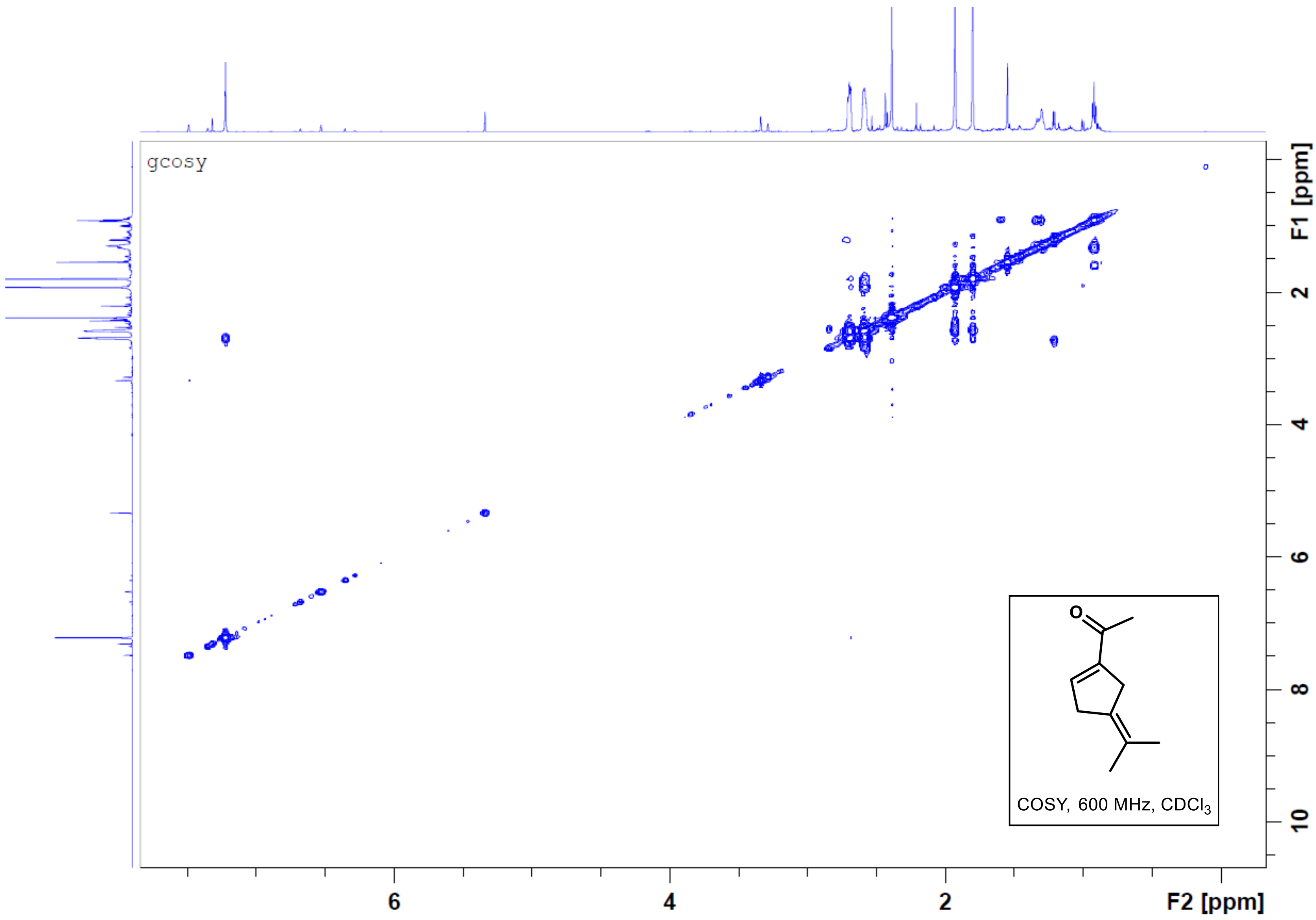
gcosy
F1 [ppm]
F2 [ppm]
COSY, 600 MHz, $CDCl_3$
O

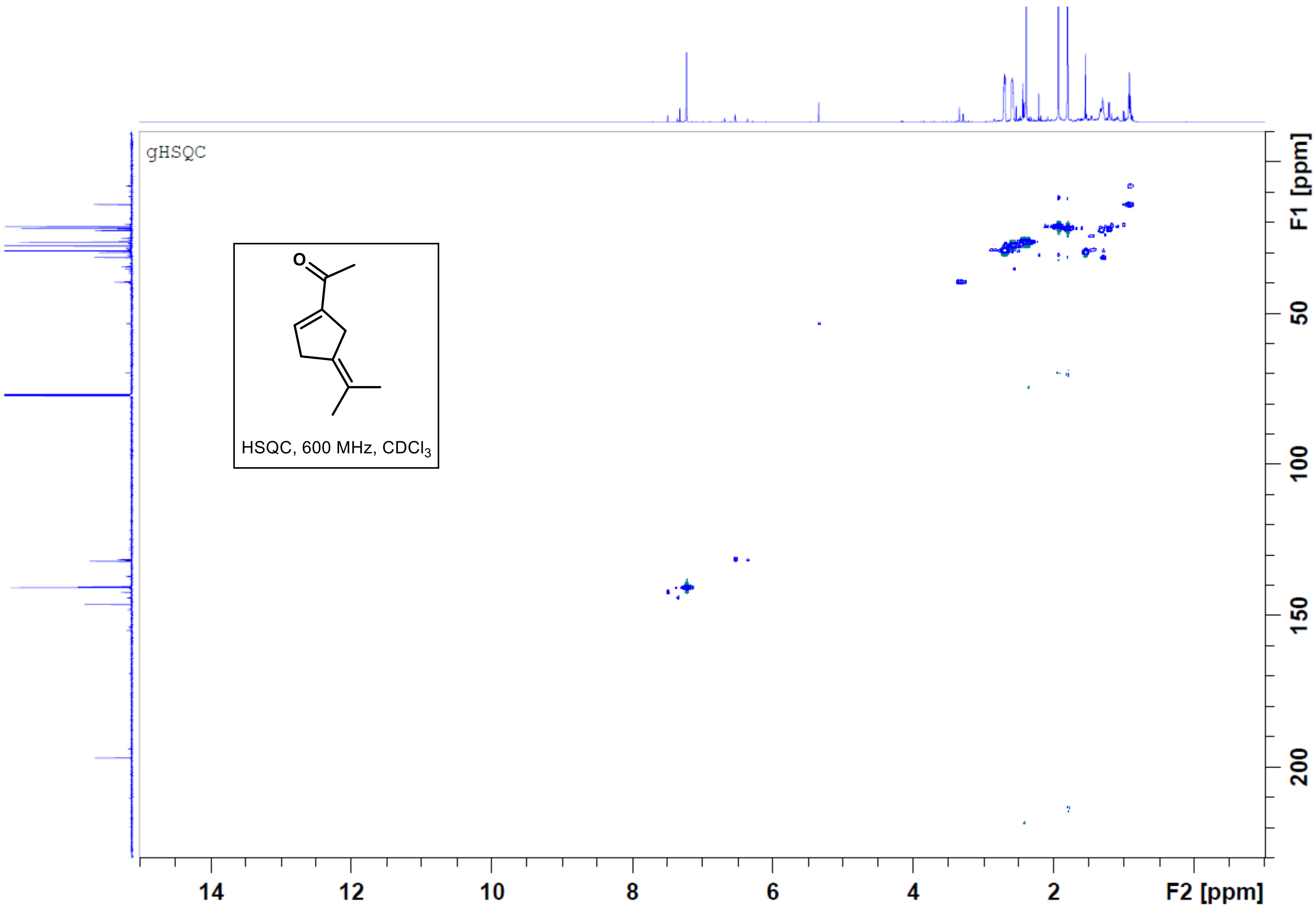
gHSQC
O
HSQC, 600 MHz, CDCl3
F1 [ppm]
50
100
150
200
14
12
10
8
6
4
2
F2 [ppm]

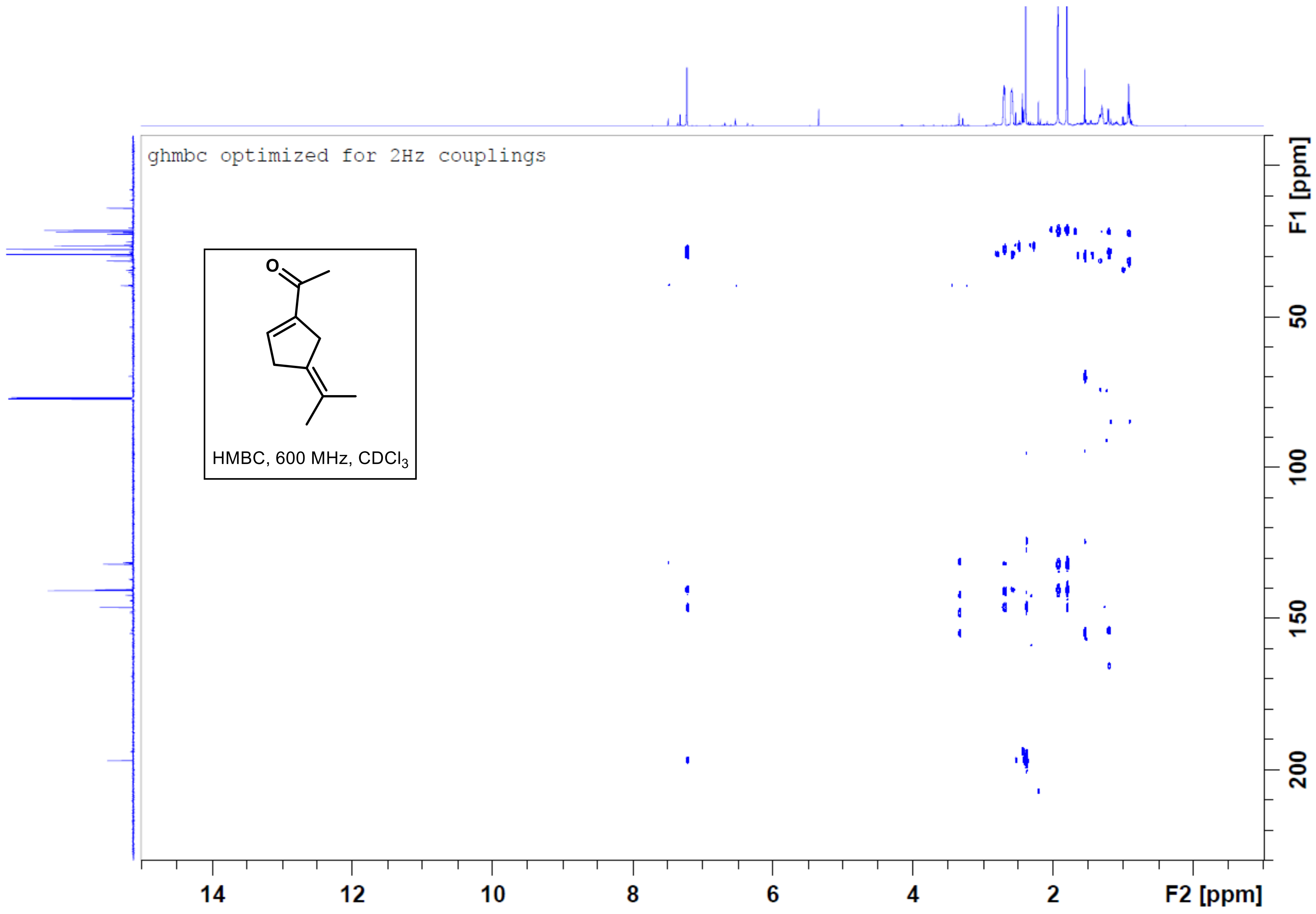
ghmbc optimized for 2Hz couplings
O
HMBC, 600 MHz, CDCl3
F1 [ppm]
50
100
150
200
14
12
10
8
6
4
2
F2 [ppm]

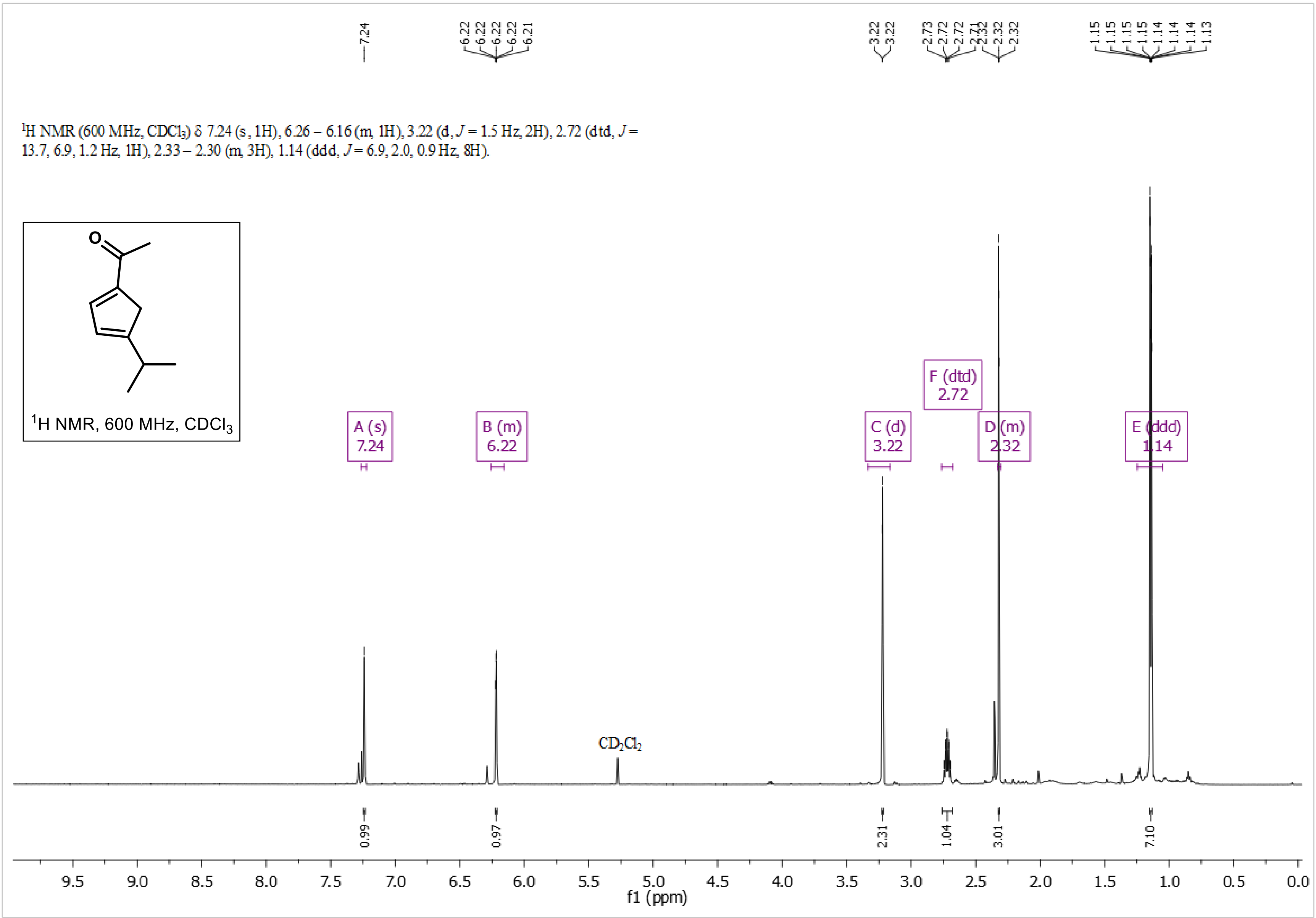
7.24
6.22
6.22
6.22
6.22
6.21
3.22
3.22
2.73
2.72
2.72
2.71
2.32
2.32
2.32
1.15
1.15
1.15
1.15
1.14
1.14
1.14
1.13
^{1}H NMR (600 MHz, $CDCl_3$) δ 7.24 (s, 1H), 6.26 – 6.16 (m, 1H), 3.22 (d, J = 1.5 Hz, 2H), 2.72 (dtd, J = 13.7, 6.9, 1.2 Hz, 1H), 2.33 – 2.30 (m, 3H), 1.14 (ddd, J = 6.9, 2.0, 0.9 Hz, 8H).
O
^{1}H NMR, 600 MHz, $CDCl_3$
A (s) 7.24
B (m) 6.22
C (d) 3.22
F (dtd) 2.72
D (m) 2.32
E (ddd) 1.14
CD_2Cl_2
0.99
0.97
2.31
1.04
3.01
7.10
9.5
9.0
8.5
8.0
7.5
7.0
6.5
6.0
5.5
5.0
4.5
4.0
3.5
3.0
2.5
2.0
1.5
1.0
0.5
0.0
f1 (ppm)

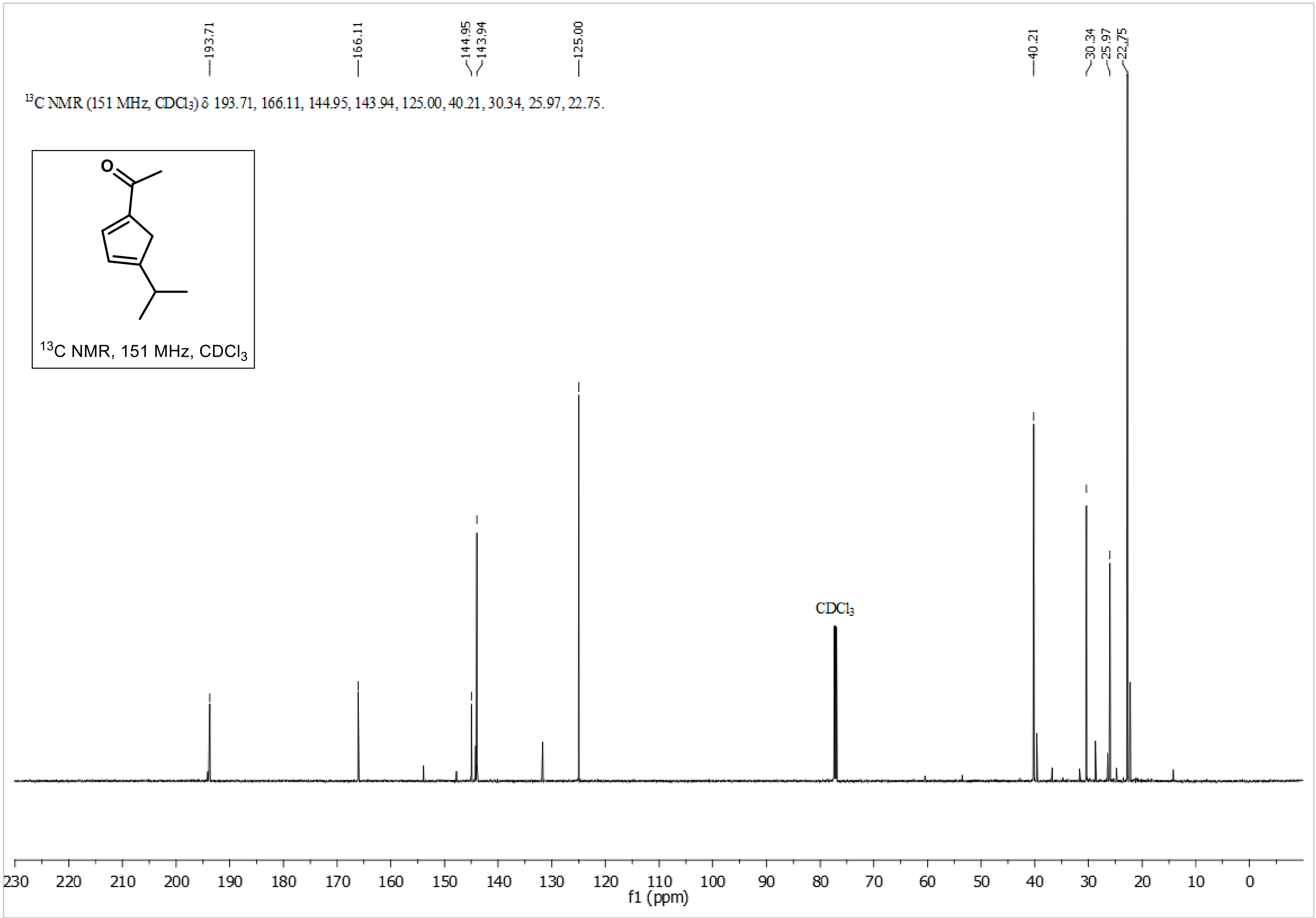

193.71
166.11
144.95
143.94
125.00
40.21
30.34
25.97
22.75
13C NMR (151 MHz, CDCl3) δ 193.71, 166.11, 144.95, 143.94, 125.00, 40.21, 30.34, 25.97, 22.75.
O
13C NMR, 151 MHz, CDCl3
CDCl3
230 220 210 200 190 180 170 160 150 140 130 120 110 100 90 80 70 60 50 40 30 20 10 0
f1 (ppm)

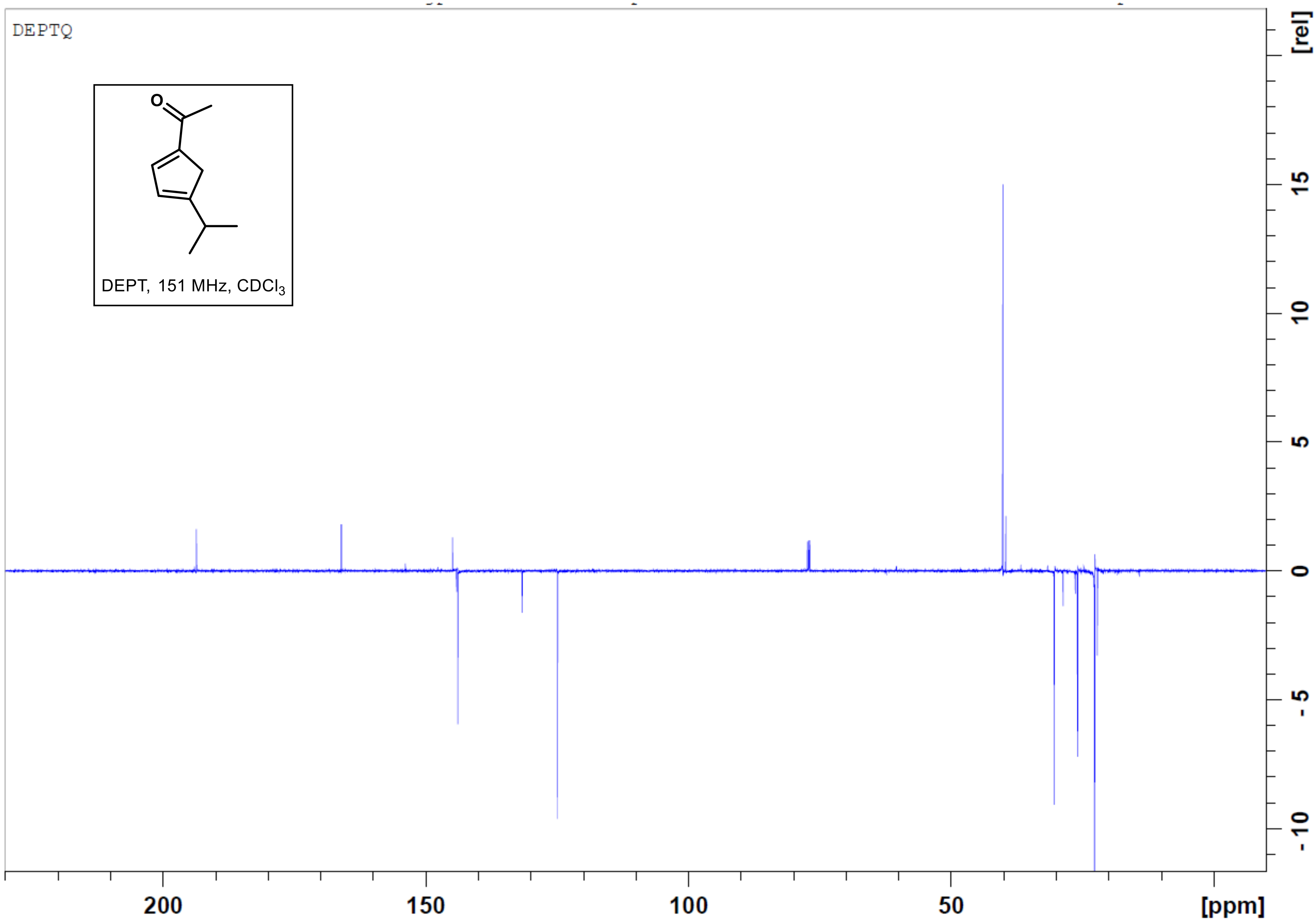

DEPTQ
O
DEPT, 151 MHz, CDCl3
200
150
100
50
[ppm]
[rel]
15
10
5
0
-5
-10

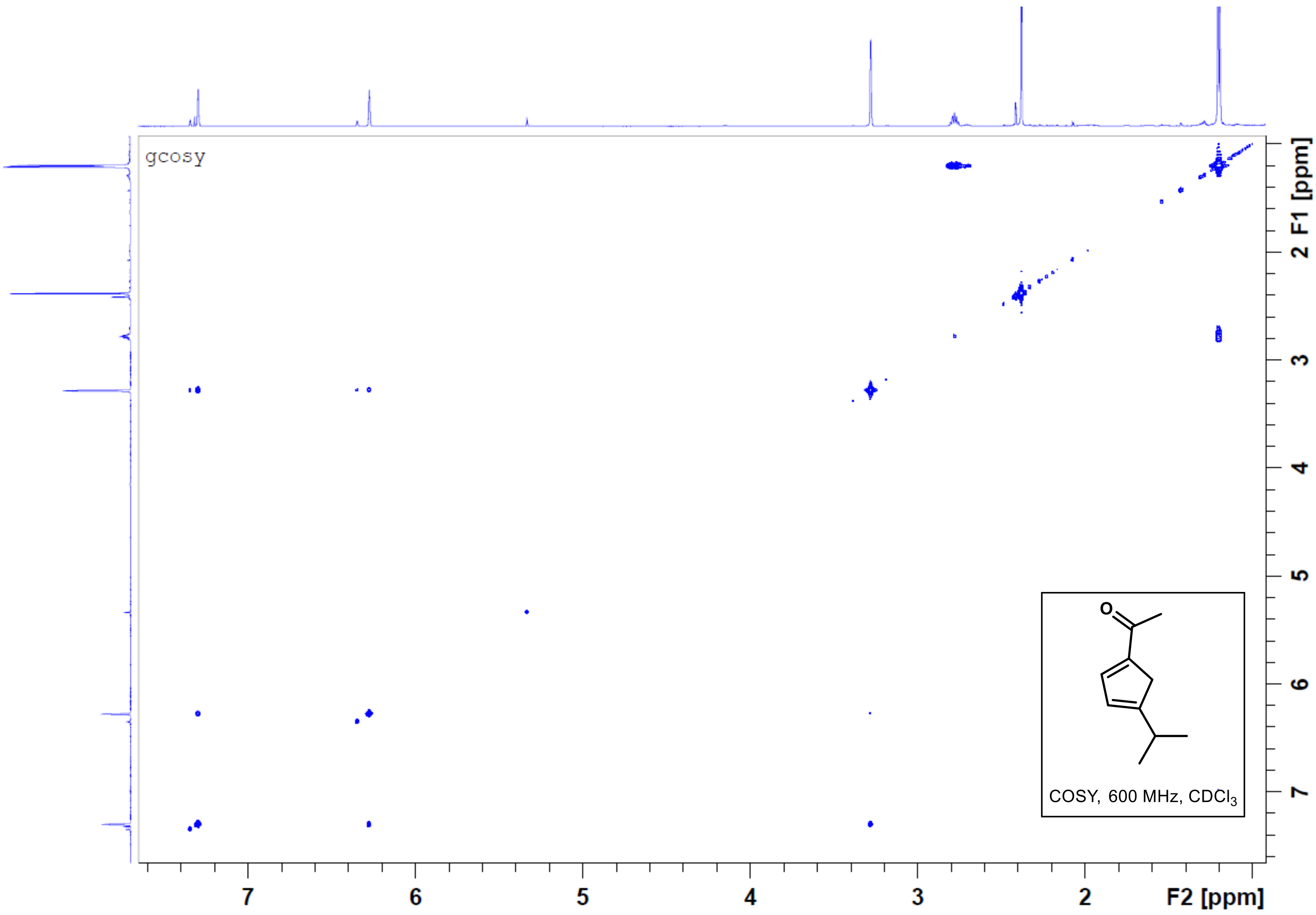
gcosy
F1 [ppm]
F2 [ppm]
O
COSY, 600 MHz, $CDCl_3$

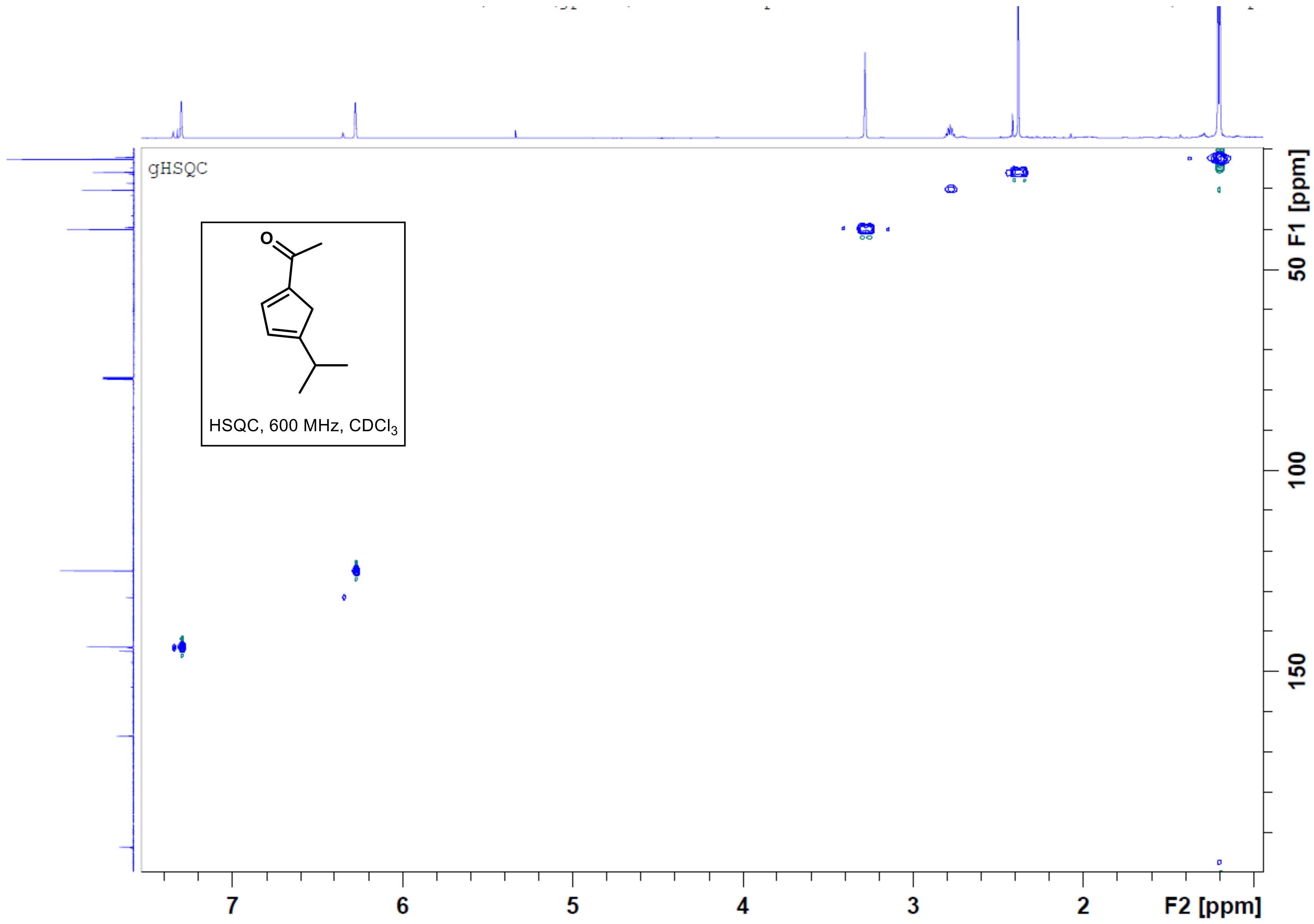
gHSQC
O
HSQC, 600 MHz, CDCl3
7
6
5
4
3
2
F2 [ppm]
50
100
150
F1 [ppm]

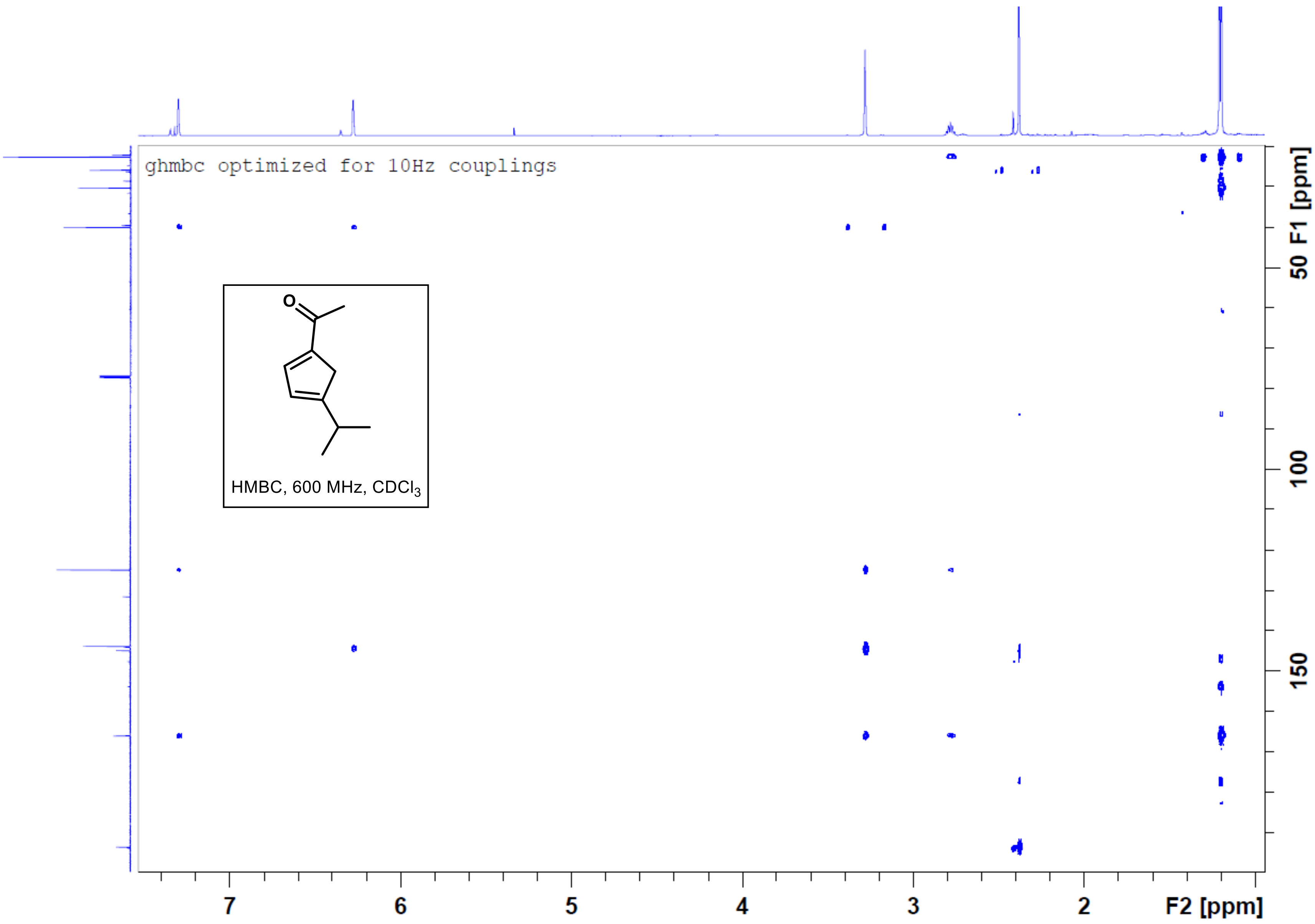
ghmbc optimized for 10Hz couplings
O
HMBC, 600 MHz, $CDCl_3$
50 F1 [ppm]
100
150
7
6
5
4
3
2
F2 [ppm]

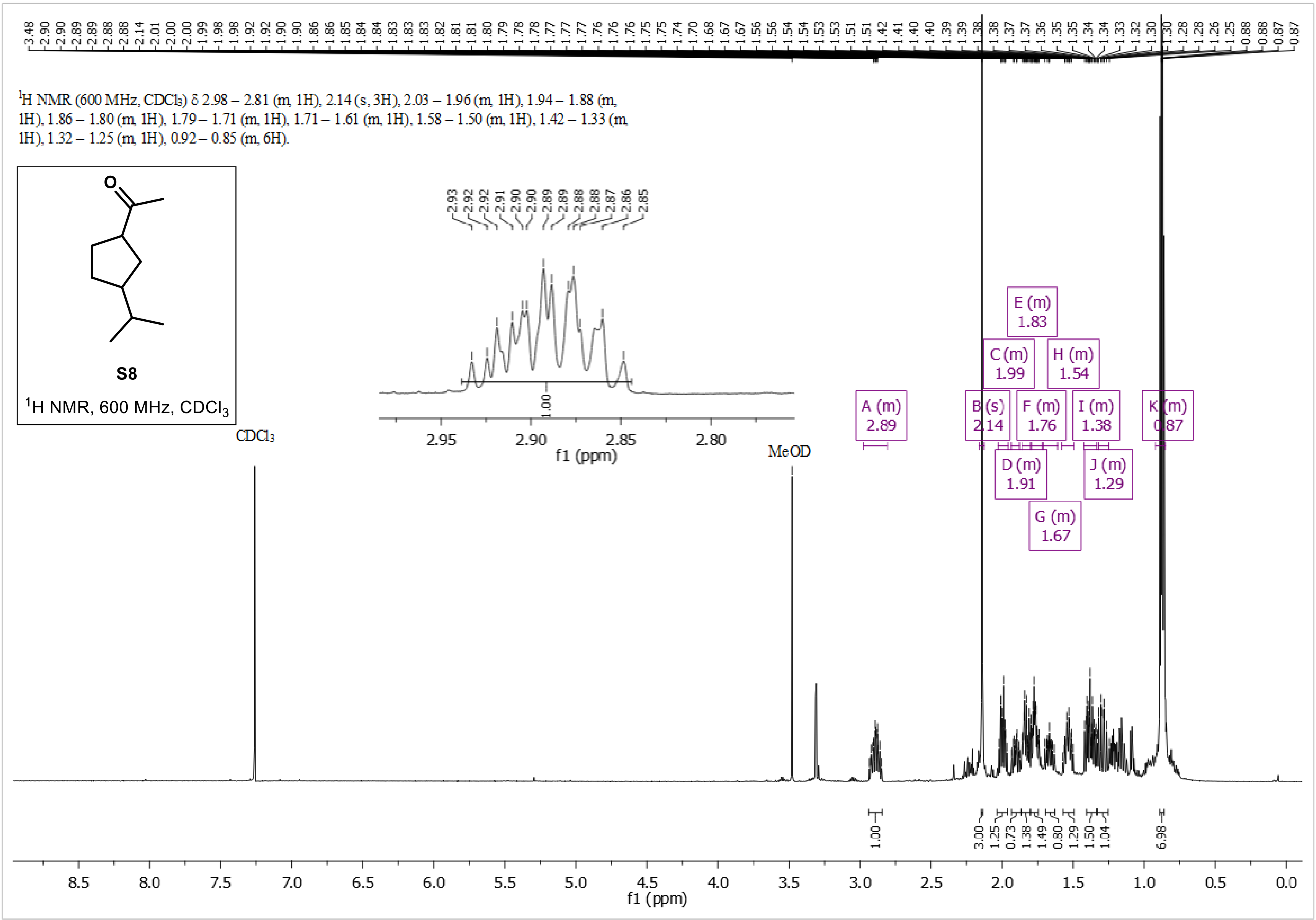

¹H NMR (600 MHz, CDCl₃) δ 2.98 – 2.81 (m, 1H), 2.14 (s, 3H), 2.03 – 1.96 (m, 1H), 1.94 – 1.88 (m, 1H), 1.86 – 1.80 (m, 1H), 1.79 – 1.71 (m, 1H), 1.71 – 1.61 (m, 1H), 1.58 – 1.50 (m, 1H), 1.42 – 1.33 (m, 1H), 1.32 – 1.25 (m, 1H), 0.92 – 0.85 (m, 6H).
O
S8
¹H NMR, 600 MHz, CDCl₃
CDCl₃
MeOD
A (m) 2.89
B (s) 2.14
C (m) 1.99
D (m) 1.91
E (m) 1.83
F (m) 1.76
G (m) 1.67
H (m) 1.54
I (m) 1.38
J (m) 1.29
K (m) 0.87
f1 (ppm)

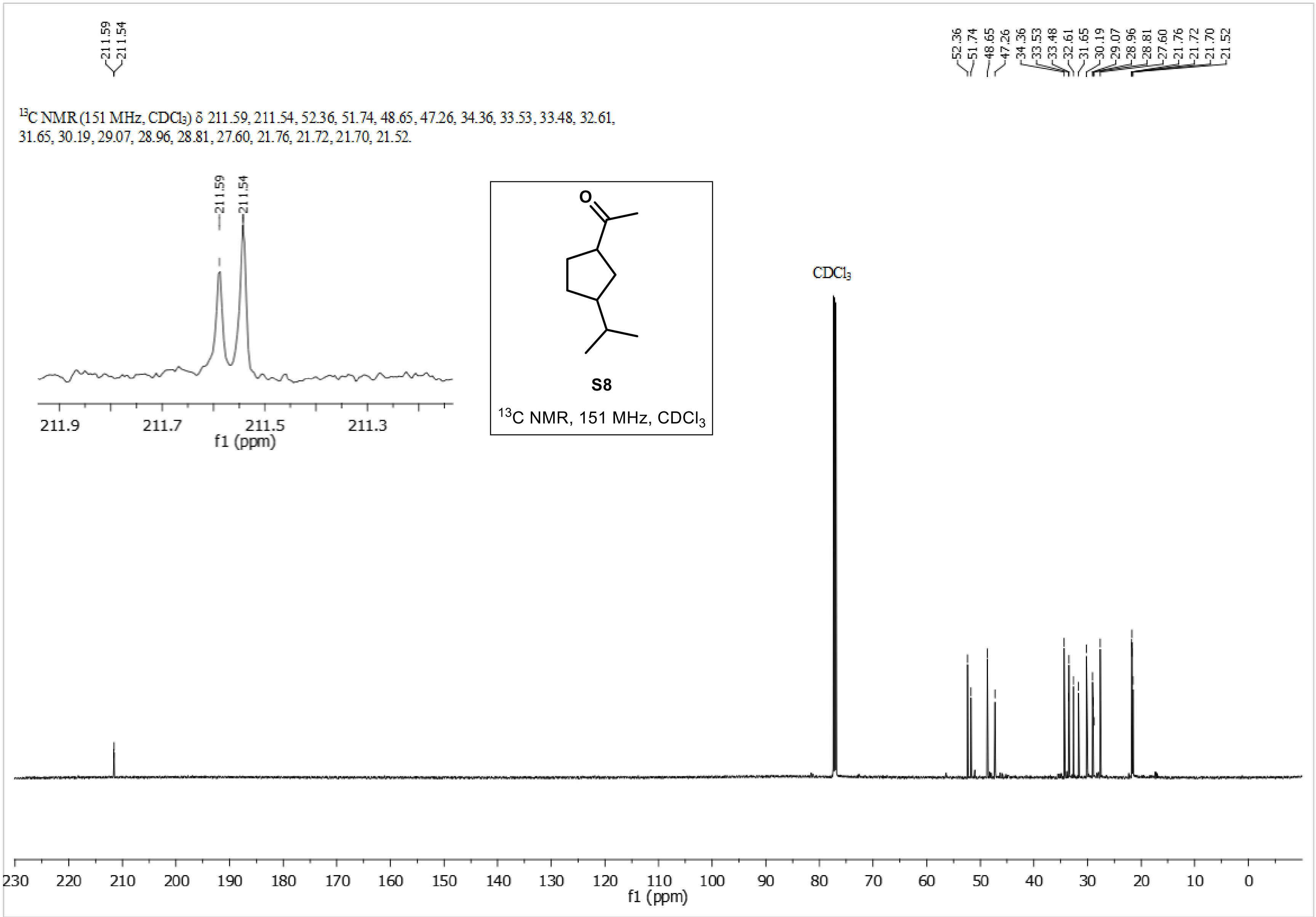
211.59
211.54
52.36
51.74
48.65
47.26
34.36
33.53
33.48
32.61
31.65
30.19
29.07
28.96
28.81
27.60
21.76
21.72
21.70
21.52
^{13}C NMR (151 MHz, $CDCl_3$) δ 211.59, 211.54, 52.36, 51.74, 48.65, 47.26, 34.36, 33.53, 33.48, 32.61, 31.65, 30.19, 29.07, 28.96, 28.81, 27.60, 21.76, 21.72, 21.70, 21.52.
211.9
211.7
211.5
211.3
f1 (ppm)
O
S8
^{13}C NMR, 151 MHz, $CDCl_3$
$CDCl_3$
230 220 210 200 190 180 170 160 150 140 130 120 110 100 90 80 70 60 50 40 30 20 10 0
f1 (ppm)
